# Crystal structures of the water and acetone monosolvates of bis­[4′-(pyridin-4-yl)-2,2′:6′,2′′-terpyridine]­manganese(II) bis­(hexa­fluorido­phosphate)

**DOI:** 10.1107/S2056989015003503

**Published:** 2015-03-04

**Authors:** Leandro M. O. Lourenço, Filipe A. Almeida Paz, José A. Fernandes

**Affiliations:** a CICECO, Chemistry Department, Universidade de Aveiro, Campus Universitário de Santiago, 3810-193 Aveiro, Portugal

**Keywords:** crystal structure, metalloligand, manganese, terpyridine

## Abstract

The crystal structures of bis­[4′-(pyridin-4-yl)-2,2′:6′,2′′-terpyridine]­man­gan­ese(II) bis­(hexa­fluorido­phosphate) monohydrate, [Mn(C_20_H_14_N_4_)_2_](PF_6_)_2_·H_2_O, (1), and bis­[4′-(pyridin-4-yl)-2,2′:6′,2′′-terpyridine]­manganese(II) bis(hexa­fluorido­phosphate) acetone monosolvate, (2), [Mn(C_20_H_14_N_4_)_2_](PF_6_)_2_·CH_3_COCH_3_, are described. At 150 K, (1) and (2) have monoclinic (*P*2_1_/*c*) and ortho­rhom­bic (*C*222_1_) symmetries, respectively. Both structures exhibit octahedrally coordinated Mn^II^ atoms and disorder. They display weak inter­actions, such as C—H⋯F, C—H⋯N, C—H⋯π, F⋯π and π–π. The twofold rotation axis in the molecule of (2) is coincident with a twofold rotation axis of the crystal.

## Abstract 2

The structure of the water and acetone solvates of bis­[4′-(pyridin-4-yl)-2,2′:6′,2′′-terpyridine]­manganese(II) bis­(hexa­fluorido­phosphate) were obtained. The compounds crystallize in the monoclinic *P*2_1_/*c* and ortho­rhom­bic *C*222_1_ space groups, respectively.

## Chemical context   

The synthesis of new metal–organic frameworks (MOFs) can be achieved by several ways with different degrees of reaction control. One way of having a tighter control on the reactions is the use of metalloligands. A metalloligand is a kind of ligand in which the bonding capabilities of the ligand are combined with the directionallity of a metal centre (Halper *et al.*, 2006 ▸; Kitagawa *et al.*, 2006 ▸; Noro *et al.*, 2005 ▸).
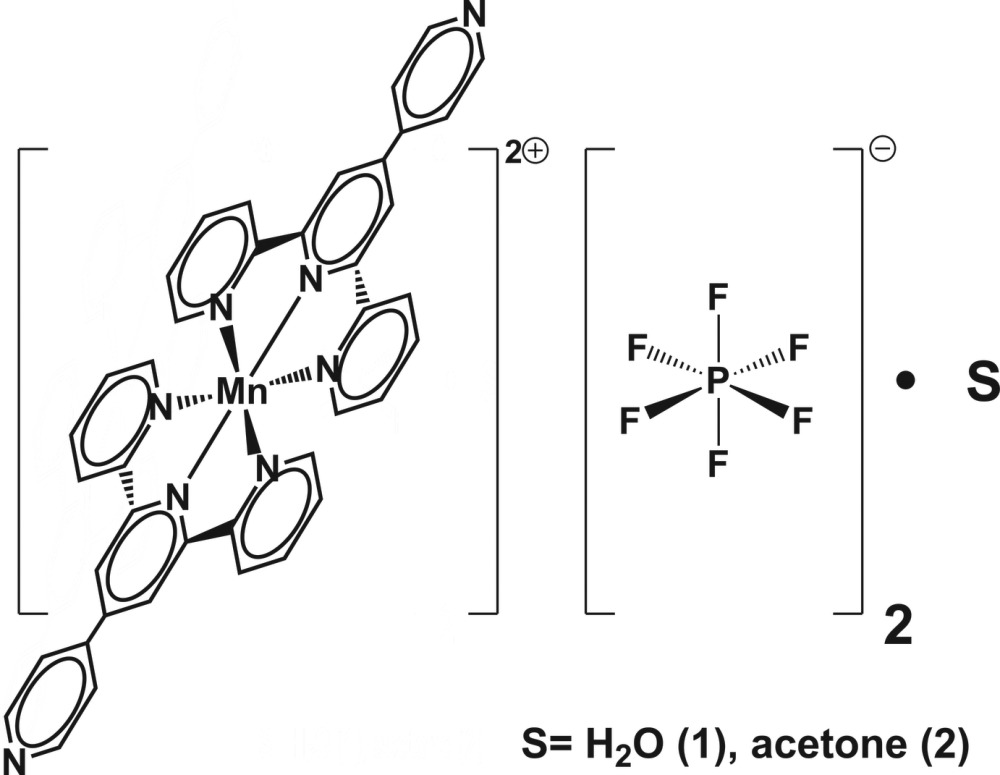


As it is found from a database survey (dedicated section below), there is already a considerable number of compounds of the type [*M*(Pyterpy)_2_^*n*+^] [Pyterpy = 4′-(pyridin-4-yl)-2,2′:6′,2′′-terpyridine]. However, no equivalent structure is found with Mn^2+^ as metallic centre. In order to fill this gap, we describe in the present report the crystal structure of the water and acetone monosolvates of bis­[4′-(pyridin-4-yl)-2,2′:6′,2′′-terpyridine]­manganese(II) bis­(hexa­fluorido­phosphate).

## Bis[4′-(pyridin-4-yl)-2,2′:6′,2′′-terpyridine]­man­gan­ese(II) bis­(hexa­fluorido­phosphate) monohydrate, (1)   

### Structural commentary   

**Figure 1:**
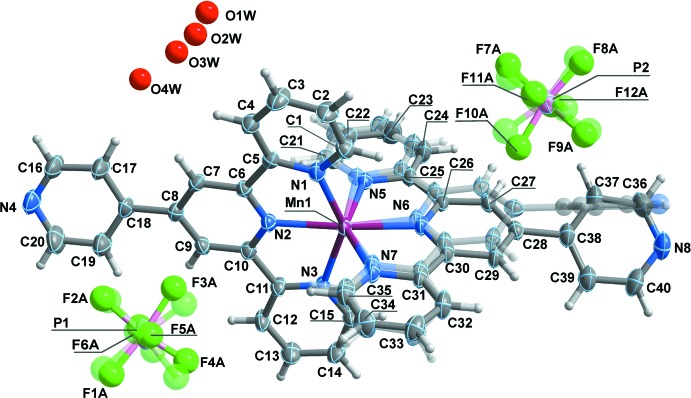
View of the molecular species present in the crystal of (1). Displacement ellipsoids are drawn at the 50% probability level and the atomic labelling is provided for all non-H atoms of the components with highest occupancies. Non-H atoms represented by spheres were isotropically refined and H atoms are depicted by spheres with arbitrary radius. The componenents with least occupancies are not numbered for the sake of clarity and represented as transparent.

The asymmetric unit of (1) (Fig. 1 ▸) comprises a dicationic coordination complex, two highly disordered hexa­fluorido­phosphate charge-balancing anions, and a water mol­ecule distributed among four general locations. The Mn^2+^ cation is distorted octahedrally coordinated by two κ^3^ *N-*(4′-(pyridin-4-yl)-2,2′:6′,2′′-terpyridine) ligands. One of these ligands is spatially disordered over two close, but distinct, locations, with a distribution of occupancies of 0.85:0.15. Concerning only the major contributor for the disordered ligand, we may say that the coordination environment around the metal cation resembles a highly distorted octa­hedron with the Mn—N distances in the 2.193 (4)–2.268 (4) Å range, and the *cis* and *trans* octa­hedral angles found in the inter­vals 71.99 (17)–117.68 (17) and 143.36 (15)–169.95 (18)°, respectively. The angle between the medium planes of the terpyridine moieties is 85.76 (14)°, and the angles between the medium planes of non-coordinating pyridines and the terpyridine to which they are attached are 7.9 (2) and 47.1 (3)° (see Table 1 ▸ for details).

### Supra­molecular features   

**Figure 2:**
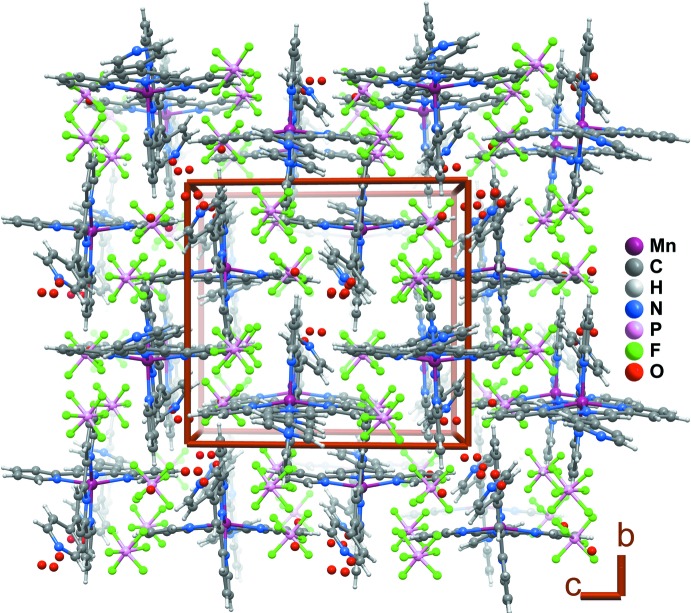
The crystal packing of (1) along the [100] direction. The components for the disordered organic ligand and hexa­fluorido­phosphate with the lowest occupation factors are not represented. Supra­molecular inter­actions are not represented for clarity.

The structure of (1) is highly disordered, and the H atoms belonging to water mol­ecules were not found. For this reason, the description of this structure cannot be thorough (see Fig. 2 ▸ for crystal packing). Nevertheless, many weak inter­actions are present in the crystal structure, namely C—H⋯F, C—H⋯N, C—H⋯π, F⋯π and π–π inter­actions (see Tables 2 ▸ and 3 ▸ for details). Additionally, there is a close relation between the disordered water mol­ecules and the entities in their neighbourhoods. While the position of site O3*W* (occupancy 0.15) is incompatible with the position of N8 (occupancy 0.85) at 2.43 (3) Å, it is foreseeable that it donates a proton to N108 at 2.94 (3) Å. The position of O4*W* (occupancy 0.85) is also incompatible with the H123 position [occupancy 0.15, distance 1.95 (5) Å]. Other contact distances are in the limit of possible hydrogen-bonding interactions, namely O1*W*⋯F6*B* [occupancies 0.4 and 0.3333, distance 2.57 (2) Å], O4*W*⋯F3*A* [occupancies 0.1 and 0.6667, distance 2.63 (5) Å] and O4*W*⋯H23 [occupancies 0.1 and 0.85, distance 2.35 (5) Å]. These relations suggest the water mol­ecule accomodates in the empty spaces left by the disordered organic moieties, or *vice versa*.

## Bis[4′-(pyridin-4-yl)-2,2′:6′,2′′-terpyridine]­manganese(II) bis­(hexa­fluorido­phosphate) acetone monosolvate, (2)   

### Structural commentary   

**Figure 3:**
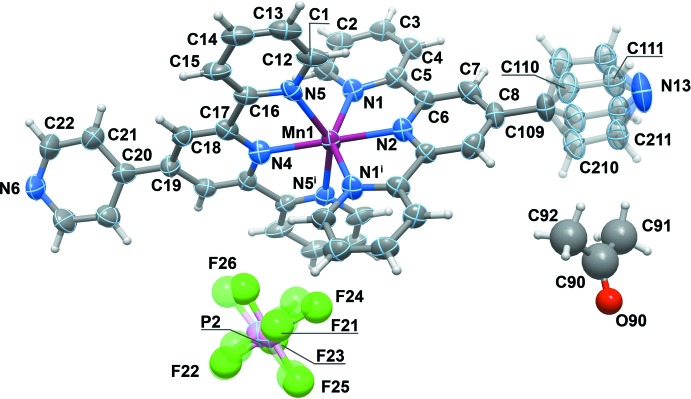
A view of the molecular species present in the crystal of (2). Displacement ellipsoids are drawn at the 50% probability level and the atomic labelling is provided for all non-H atoms of the components with highest occupancies. Non-H atoms represented by spheres were isotropically refined and H atoms are depicted by spheres with arbitrary radius. The componenents with least occupancies are not numbered for the sake of clarity and represented as transparent.

Except for the type of the co-crystallizing solvent, compound (2) (Fig. 3 ▸) is very similar to (1). However, the molecule of (2) exhibits a twofold rotation axis which is coincident to the axis of the coordination complex, which passes through atoms N13, C109, C8, N2, Mn1, N4, C19, C20 and N6. Thus, the asymmetric unit comprises one half of the dicationic coordination compound, one disordered charge balancing hexa­fluorido­phosphate anion and half of an acetone mol­ecule. In the cation, only one of the noncoordinating pyridine moieties is affected by disorder. Nevertheless, the geometrical environment around the metal cation is still a distorted octa­hedron (with a symmetry axis in one of the diagonals), with Mn—N distances in the 2.180 (13)–2.247 (11) Å range, and the *cis* and *trans* octa­hedral angles in the inter­vals 72.3 (3)–107.7 (5) and 144.7 (5)–180.0°, respectively. The angle between the medium planes of the terpyridine moieties is 89.5 (3)°, and the angles between the medium planes of non-coordinating pyridines and the terpyridine to which they are attached are 35.3 (non-­disordered), 62.3 (12) and 65.8 (13)° (disordered) (see Table 4 ▸ for details).

### Supra­molecular features   

**Figure 4:**
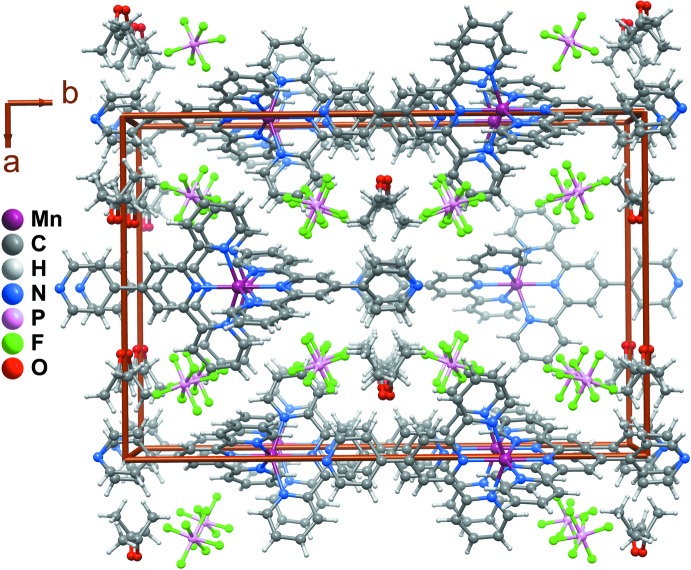
The crystal packing of (2) along the [001] direction. The components for the disordered organic ligand and hexa­fluorido­phosphate with the lowest occupation factors are not represented. Supra­molecular inter­actions are not represented for clarity.

Similarly to (1), in the structure of compound (2) (Fig. 4 ▸) there are a considerable number of weak inter­actions present, namely C—H⋯F, C—H⋯O, C—H⋯π, F⋯π and π–π inter­actions (see Tables 5 ▸ and 6 ▸ for details).

## Database survey   

The use of the ligand Pyterpy [described by Constable *et al.* (2000 ▸)] as precursor of metalloligands has contributed for the synthesis of several complexes of the form [*M*(Pyterpy)_2_]^*n*+^(*X* ^−^)_*n*_. The metal centres comprise metals with valence +2 of the first transition period, from Fe^2+^ to Zn^2+^, as well as Co^3+^, Ru^2+^ and Rh^3+^ (Groom & Allen, 2014 ▸). The charge-balancing anions are PF_6_^−^, NO_3_^−^, ClO_4_^−^, SCN^−^ or [Fe(SCN)_6_]^3−^ (Beves, Bray *et al.*, 2008 ▸; Beves, Constable, Housecroft, Kepert, Neuburger *et al.*, 2007 ▸; Beves, Dunphy *et al.*, 2008 ▸; Constable *et al.*, 2000 ▸, 2006 ▸; Ding *et al.*, 2009 ▸; Indumathy *et al.*, 2007 ▸; Mehrani *et al.*, 2013 ▸; Morsali *et al.*, 2009 ▸; Paul *et al.*, 2004 ▸; Pitarch López *et al.*, 2005 ▸; see Table 7 ▸ for details).

All the cations exhibit a distorted octa­hedral geometry, with the Pyterpy ligands in a meridional coordination. Some of the crystal structures sharing the same anion are isotypical. This is the case of the nitrate-containing crystals of Fe^2+^, Co^2+^ and Ni^2+^ [Cambridge Structural Database (CSD; Groom & Allen, 2014 ▸) refcodes WOMXAX, VEYGIQ and OFUJUV] or the hexa­fluorido­phosphate-containing crystals of Fe^2+^ and Ru^2+^ (OFUKEG and KITFEZ). Two different solvates of [Fe(Pyterpy)(PyterpyH)][Fe(SCN)_6_] are also isotypical (XIQFIN and XIQFEJ). Additionally, two structures of Fe^2+^ and Ru^2+^ have similar cell parameters, despite of not sharing the same anion (OFUKIK and UGEKEX). None of compounds described in this work is isotypical with a previously reported structure.

Until now the use of the metalloligand [*M*(Pyterpy)_2_^*n*+^] is still very limited. Some one-dimensional polymers are known (Beves, Constable *et al.*, 2008 ▸; Yoshida *et al.*, 2009 ▸; Beves, Constable, Housecroft, Kepert, Price *et al.*, 2007 ▸). Among the oligomers we can find linear structures with three (Liu *et al.*, 2014 ▸) or five metal coordination centres (Beves *et al.*, 2009 ▸) and hexa­nuclear cyclic clusters (Liu *et al.*, 2014 ▸; see Table 8 ▸ for details).

## Synthesis and crystallization   

All the reactants were purchased from commercial suppliers and used as received.

### 4′-(Pyridin-4-yl)-2,2′:6′,2′′-terpyridine   

The ligand Pyterpy was synthesized by a mechanochemical reaction of 2-acetyl­pyridine, 4-pyridine­carboxaldehyde and NaOH, followed by refluxing with ammonium acetate in acetic acid for 24 h (Cave & Raston, 2001 ▸).

### Title compounds   

A solution of Pyterpy (132.7 mg, 0.42 mmol) in MeOH (50 mL) was added dropwise to a solution of [Mn(CH_3_COO)_2_]·4H_2_O (52.4 mg, 0.21 mmol) in 5 mL of water. The mixture refluxed at 338 K overnight to obtain a complete reaction. After this period, the solution was concentrated until a light-brown solid was obtained. The solid was filtrated and washed with water and ethanol to remove the impurities. The solid was dried at 333 K. Analysis calculated for [C_40_H_28_F_12_MnN_8_][PF_6_]_2_·H_2_O: C 47.97, H 3.22, N 11.19%; found: C 47.37, H 3.19, N 11.09%.

Suitable crystals for X-ray diffraction were obtained by diffusion of water into a solution of the title compound in acetone. Two types of crystals were harvested corresponding to two different solvates.

## Refinement   

Crystal data, data collection and structure refinement details are summarized in Table 9 ▸.

H atoms bound to carbon were placed at their idealized positions and were included in the final structural model in riding-motion approximation, with C—H = 0.95 Å (aromatic C—H) or C—H = 0.98 Å (aliphatic C—H). The isotropic displacement parameters for these atoms were fixed at 1.2 times *U*_eq_ of the respective parent carbon atom. Some parts of the two crystal structures are subjected to spatial disorder.

In (1), the disorder affects one whole ligand which is placed over two close, but not coincident locations, with occupancies 0.85:0.15. The two crystallographically independent PF_6_^−^ anions are distributed over four distinct orientations with coincidence of the central P atoms, and occupancies 2/3:1/3, 2/3:1/3. The P atoms were refined anisotropically, and the F atoms isotropically with a common *U*_iso_. The water mol­ecule of crystallization was distributed over four distinct locations, which were isotropically refined with a common *U*_iso_, and total occupancy equal to 1. The H atoms of the solvent were not located, but were added in the formula unit.

In (2), the disorder in the organic ligand was limited to a terminal 4-pyridine moiety, which was refined anisotropically over two locations with equal occupancies. The sole PF_6_^−^ in (2) was distributed among two locations with occupancies 0.6:0.4 with P-atoms not coincident in space. The overall quality of the crystal was not sufficient for a precise determination of the Flack parameter.

## Supplementary Material

Crystal structure: contains datablock(s) 1, 2. DOI: 10.1107/S2056989015003503/hg5428sup1.cif

Structure factors: contains datablock(s) 1. DOI: 10.1107/S2056989015003503/hg54281sup2.hkl

Structure factors: contains datablock(s) 2. DOI: 10.1107/S2056989015003503/hg54282sup3.hkl

CCDC references: 1050540, 1050539

Additional supporting information:  crystallographic information; 3D view; checkCIF report

## Figures and Tables

**Table 1:** Selected geometric parameters (Å, °) for (1)

Mn1—N1	2.231 (4)	Mn1—N5	2.259 (5)
Mn1—N2	2.193 (4)	Mn1—N6	2.212 (4)
Mn1—N3	2.268 (4)	Mn1—N7	2.260 (5)
			
N1—Mn1—N2	72.37 (15)	N2—Mn1—N7	117.68 (17)
N1—Mn1—N3	143.36 (15)	N3—Mn1—N5	94.0 (3)
N1—Mn1—N5	99.8 (3)	N3—Mn1—N6	105.2 (3)
N1—Mn1—N6	111.3 (3)	N3—Mn1—N7	96.0 (4)
N1—Mn1—N7	92.2 (4)	N5—Mn1—N6	72.45 (17)
N2—Mn1—N3	72.21 (15)	N5—Mn1—N7	144.44 (17)
N2—Mn1—N5	97.87 (16)	N6—Mn1—N7	71.99 (17)
N2—Mn1—N6	169.95 (18)		

**Table 2:** Hydrogen-bond geometry (Å, °) for (1)

*D*—H⋯*A*	*D*—H	H⋯*A*	*D*⋯*A*	*D*—H⋯*A*
C12—H12⋯F5*A* ^i^	0.95	2.50	3.422 (9)	164
C15—H15⋯F12*A* ^ii^	0.95	2.46	3.305 (9)	149
C16—H16⋯F7*A* ^iii^	0.95	2.38	3.289 (9)	160
C19—H19⋯F5*A* ^i^	0.95	2.41	3.328 (10)	162
C29—H29⋯N4^iv^	0.95	2.35	3.270 (8)	163

Symmetry codes: (i) 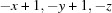; (ii) 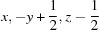; (iii) 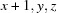; (iv) 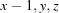.

**Table 3:** Intra­molecular contacts (Å, °) for (1)

*D*—*X*⋯A	*X*⋯A	*D*—*X*⋯A
C13—H13⋯*Cg*1^vi^	2.83	152
P1*A*—F5*A*⋯*Cg*2	3.108 (8)	129.0 (4)
P2*A*—F12*A*⋯*Cg*3^vii^	2.906 (9)	131.0 (4)
		
*Cg*⋯*Cg*	*Cg*⋯*Cg*	
*Cg*4⋯*Cg*5	3.779 (3)	
*Cg*5⋯*Cg*6^i^	3.778 (3)	

Symmetry codes: (i) −*x* + 1, −*y* + 1, −*z*; (vi) *x*, 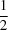 − *y*, −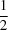 + *z*; (vii) *x*, 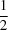 − *y*, 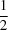 + *z. Cg*1: centroid of {N5, C21—C25}; *Cg*2: centroid of {N2, C6—C10}; *Cg*3: centroid of {N6, C26—C30}; *Cg*4: centroid of {N1, C1—C5}; *Cg*5: centroid of {N3, C11—C15}; *Cg*6: centroid of {N4, C16—C20}.

**Table 4:** Selected geometric parameters (Å, °) for (2)

Mn1—N1	2.210 (10)	Mn1—N4	2.187 (14)
Mn1—N2	2.180 (13)	Mn1—N5	2.247 (11)
			
N1—Mn1—N1^i^	144.8 (5)	N2—Mn1—N4	180.0
N1—Mn1—N2	72.4 (3)	N2—Mn1—N5	107.7 (3)
N1—Mn1—N4	107.6 (3)	N4—Mn1—N5	72.3 (3)
N1—Mn1—N5	93.5 (4)	N5—Mn1—N5^i^	144.7 (5)
N1—Mn1—N5^i^	97.0 (4)		

Symmetry code: (i) 
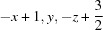
.

**Table 5:** Hydrogen-bond geometry (Å, °) for (2)

*D*—H⋯*A*	*D*—H	H⋯*A*	*D*⋯*A*	*D*—H⋯*A*
C12—H12⋯F25^ii^	0.95	2.31	3.23 (3)	162
C15—H15⋯O90^ii^	0.95	2.58	3.53 (3)	177
C18—H18⋯O90^iii^	0.95	2.57	3.50 (2)	168
C18—H18⋯O90^ii^	0.95	2.53	3.47 (2)	169
C22—H22⋯F21^iv^	0.95	2.47	3.42 (2)	175

Symmetry codes: (ii) 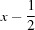, 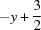, 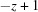; (iii) 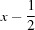, 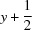, 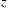; (iv) 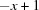, 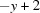, 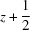.

**Table 6:** Intra­molecular contacts (Å, °) for (2)

*D*—*X*⋯A	*X*⋯A	*D*—*X*⋯A
C2—H2⋯*Cg*1^v^	2.72	156
P2—F25⋯*Cg*2^vi^	3.091 (18)	153.0 (10)
		
*Cg*⋯*Cg*	*Cg*⋯*Cg*	
*Cg*3⋯*Cg*3^vii^	3.539 (7)	

Symmetry codes: (v) *x*, *y*, *z* + 1; (vi) −*x* + 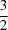, −*y* + 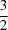, *z* − 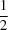; (vii) −*x* + 1, *y*, −*z* + 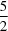. *Cg*1: centroid of {N5, C12—C16}; *Cg*2: centroid of {N2, C6—C8, C6^i^, C7^i^}; *Cg*3: centroid of {N1, C1—C5};

**Table 7:** Known structures of the type [*M*(Pyterpy)_2_]^*n*+^(*X* ^−^)_*n*_

Metal Center	Anion	CCDC code	Reference	Space Group
Fe^2+^	PF_6_^−^	KITFEZ	Beves, Dunphy, *et al.* (2008 ▸)	*I*4_1_/*a*
Fe^2+^	ClO_4_^−^	OFUKAC	Beves, Bray *et al.* (2008 ▸)	*P* 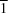
Fe^2+^	SCN^−^	UGEKEX	Morsali *et al.* (2009 ▸)	*P* 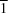
Fe^2+^	NO_3_^−^	WOMXAX	Constable *et al.* (2000 ▸)	*P* 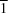
Fe^2+^	ClO_4_^−^	XIQFEJ	(*a*)	*P*2_1_/*c*
Fe^2+^	[Fe(SCN)_6_]^3−^	XIQFIN	(*a*)	*Pbcn*
Fe^2+^	[Fe(SCN)_6_]^3−^	XISWUS	(*a*)	*Pbcn*
Co^2+^	NO_3_^−^	VEYGIQ	Indumathy *et al.* (2007 ▸)	*P* 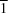
Co^3+^	ClO_4_^−^	VEYGEM	Indumathy *et al.* (2007 ▸)	*P* 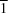
Ni^2+^	NO_3_^−^	OFUJUV	Beves, Bray *et al.* (2008 ▸)	*P* 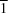
Cu^2+^	PF_6_^−^	FIYHIF	Pitarch López *et al.* (2005 ▸)	*P*4_1_
Zn^2+^	NO_3_^−^	ULAFET	Ding *et al.* (2009 ▸)	*P*4_3_
Zn^2+^	ClO_4_^−^	BIGDEC	Mehrani *et al.* (2013 ▸)	*C*2/*c*
Ru^2+^	PF_6_^−^	OFUKEG	Beves, Bray *et al.* (2008 ▸)	*I*4_1_/*a*
Ru^2+^	PF_6_^−^/NO_3_^−^	OFUKIK	Beves, Bray *et al.* (2008 ▸)	*P* 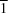
Ru^2+^	[Fe(SCN)_6_]^3−^	OFUKOQ	Beves, Bray *et al.* (2008 ▸)	*C*2/*c*
Ru^2+^	PF_6_^−^/NO_3_^−^	PEHPOI	Constable *et al.* (2006 ▸)	*P*2_1_/*c*
Rh^3+^	PF_6_^−^	DAHDOG	Paul *et al.* (2004 ▸)	*P* 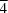 2_1_ *c*

(*a*) Beves, Constable, Housecroft, Kepert, Neuburger *et al.* (2007 ▸).

**Table 8:** Uses of [*M*(Pyterpy)_2_]^*n*+^ as metalloligand

Metal 1	Metal 2	CCDC code	Type†	Reference
Fe^2+^	Cu^2+^	GIVDEV	polymer	Beves, Constable *et al.* (2008 ▸)
Fe^2+^	Cu^2+^	OGOTEK	5 (linear)	Beves *et al.* (2009 ▸)
Ni^2+^	Co^2+^	WUTTEL	polymer	Yoshida *et al.* (2009 ▸)
Ni^2+^	Co^2+^	WUTTIP	polymer	Yoshida *et al.* (2009 ▸)
Co^2+^	Co^2+^	WUTTOP	polymer	Yoshida *et al.* (2009 ▸)
Ni^2+^	Ir^3+^	MITQUD	6 (cyclic)	Liu *et al.* (2014 ▸)
Ni^2+^	Rh^3+^	MITRAK	6 (cyclic)	Liu *et al.* (2014 ▸)
Cu^2+^	Ir^3+^	MITCEZ	6 (cyclic)	Liu *et al.* (2014 ▸)
Cu^2+^	Rh^3+^	MITCID	6 (cyclic)	Liu *et al.* (2014 ▸)
Zn^2+^	Ir^3+^	MITQEN	6 (cyclic)	Liu *et al.* (2014 ▸)
Zn^2+^	Ir^3+^	MITQIR	6 (cyclic)	Liu *et al.* (2014 ▸)
Zn^2+^	Rh^3+^	MITQOX	6 (cyclic)	Liu *et al.* (2014 ▸)
Ni^2+^	Rh^3+^	MITCOJ	3 (linear)	Liu *et al.* (2014 ▸)
Ru^2+^	Ag^+^	WICSIL	polymer	(*b*)

Notes: ‘Metal 1’ coordinated by Pyterpy; ‘Metal 2’ bridging metal. † Polymer or number of metals in the oligomer and respective arrangement; (*b*) Beves, Constable, Housecroft, Kepert, Price *et al.* (2007 ▸).

**Table 9:** Experimental details

	(1)	(2)
Crystal data		
Chemical formula	[Mn(C_20_H_14_N_4_)_2_](PF_6_)_2_·H_2_O	[Mn(C_20_H_14_N_4_)_2_](PF_6_)_2_·C_3_H_6_O
*M*_r_	983.60	1023.66
Crystal system, space group	Monoclinic, *P*2_1_/*c*	Orthorhombic, *C*222_1_
Temperature (K)	150	150
*a*, *b*, *c* (Å)	16.2389 (5), 15.3506 (5), 16.5549 (5)	18.0996 (15), 27.470 (2), 8.5734 (6)
α, β, γ (°)	90, 99.3892 (17), 90	90, 90, 90
*V* (Å^3^)	4071.5 (2)	4262.7 (6)
*Z*	4	4
Radiation type	Mo *K*α	Mo *K*α
μ (mm^−1^)	0.50	0.48
Crystal size (mm)	0.12 × 0.08 × 0.08	0.16 × 0.08 × 0.04

Data collection		
Diffractometer	Bruker APEXII CCD	Bruker APEXII CCD
Absorption correction	Multi-scan (*SADABS*; Bruker, 2007 ▸)	Multi-scan (*SADABS*; Bruker, 2007 ▸)
*T*_min_, *T*_max_	0.953, 0.960	0.955, 0.981
No. of measured, independent and observed [*I* > 2σ(*I*)] reflections	44958, 7445, 5275	18921, 3908, 2683
*R*_int_	0.085	0.091
(sin θ/λ)_max_ (Å^−1^)	0.602	0.602

Refinement		
*R*[*F* ^2^ > 2σ(*F* ^2^)], *wR*(*F* ^2^), *S*	0.089, 0.244, 1.07	0.104, 0.270, 1.07
No. of reflections	7445	3908
No. of parameters	608	292
No. of restraints	78	102
H-atom treatment	H-atom parameters constrained	H-atom parameters constrained
	*w* = 1/[σ^2^(*F*_o_^2^) + (0.1065*P*)^2^ + 15.4857*P*] where *P* = (*F*_o_^2^ + 2*F*_c_^2^)/3	*w* = 1/[σ^2^(*F*_o_^2^) + (0.0919*P*)^2^ + 48.9339*P*] where *P* = (*F*_o_^2^ + 2*F*_c_^2^)/3
Δρ_max_, Δρ_min_ (e Å^−3^)	0.98, −1.03	0.71, −1.05
Absolute structure	–	Flack *x* determined using 836 quotients [(*I* ^+^) − (*I* ^−^)]/[(*I* ^+^) + (*I* ^−^)] (Parsons *et al.* (2013 ▸)
Absolute structure parameter	–	0.21 (2)

Computer programs: *APEX2* and *SAINT* (Bruker, 2007 ▸), *SHELXS97*, *SHELXL97* and *SHELXTL* (Sheldrick, 2008 ▸), *SHELXL2014* (Sheldrick, 2015 ▸).

## supplementary crystallographic information

### (1) Bis[4'-(pyridin-4-yl)-2,2':6',2''-terpyridine]manganese(II) bis(hexafluoridophosphate) monohydrate Crystal data

**Table:**

[Mn(C_20_H_14_N_4_)_2_](PF_6_)_2_·H_2_O	*F*(000) = 1988
*M**_r_* = 983.60	*D*_x_ = 1.605 Mg m^−^^3^
Monoclinic, *P*2_1_/*c*	Mo *K*α radiation, λ = 0.71073 Å
*a* = 16.2389 (5) Å	Cell parameters from 4312 reflections
*b* = 15.3506 (5) Å	θ = 2.3–21.7°
*c* = 16.5549 (5) Å	µ = 0.50 mm^−^^1^
β = 99.3892 (17)°	*T* = 150 K
*V* = 4071.5 (2) Å^3^	Block, yellow
*Z* = 4	0.12 × 0.08 × 0.08 mm

### (1) Bis[4'-(pyridin-4-yl)-2,2':6',2''-terpyridine]manganese(II) bis(hexafluoridophosphate) monohydrate Data collection

**Table:**

Bruker APEXII CCD diffractometer	5275 reflections with *I* > 2σ(*I*)
ω and φ scans	*R*_int_ = 0.085
Absorption correction: multi-scan (SADABS; Bruker, 2007)	θ_max_ = 25.4°, θ_min_ = 2.7°
*T*_min_ = 0.953, *T*_max_ = 0.960	*h* = −19→19
44958 measured reflections	*k* = −18→17
7445 independent reflections	*l* = −19→17

### (1) Bis[4'-(pyridin-4-yl)-2,2':6',2''-terpyridine]manganese(II) bis(hexafluoridophosphate) monohydrate Refinement

**Table:**

Refinement on *F*^2^	78 restraints
Least-squares matrix: full	Hydrogen site location: inferred from neighbouring sites
*R*[*F*^2^ > 2σ(*F*^2^)] = 0.089	H-atom parameters constrained
*wR*(*F*^2^) = 0.244	*w* = 1/[σ^2^(*F*_o_^2^) + (0.1065*P*)^2^ + 15.4857*P*] where *P* = (*F*_o_^2^ + 2*F*_c_^2^)/3
*S* = 1.07	(Δ/σ)_max_ < 0.001
7445 reflections	Δρ_max_ = 0.98 e Å^−^^3^
608 parameters	Δρ_min_ = −1.03 e Å^−^^3^

### (1) Bis[4'-(pyridin-4-yl)-2,2':6',2''-terpyridine]manganese(II) bis(hexafluoridophosphate) monohydrate Special details

**Table:**

Geometry. All e.s.d.'s (except the e.s.d. in the dihedral angle between two l.s. planes) are estimated using the full covariance matrix. The cell e.s.d.'s are taken into account individually in the estimation of e.s.d.'s in distances, angles and torsion angles; correlations between e.s.d.'s in cell parameters are only used when they are defined by crystal symmetry. An approximate (isotropic) treatment of cell e.s.d.'s is used for estimating e.s.d.'s involving l.s. planes.

### (1) Bis[4'-(pyridin-4-yl)-2,2':6',2''-terpyridine]manganese(II) bis(hexafluoridophosphate) monohydrate Fractional atomic coordinates and isotropic or equivalent isotropic displacement parameters (Å^2^)

**Table:**

	*x*	*y*	*z*	*U*_iso_*/*U*_eq_	Occ. (<1)
Mn1	0.23762 (5)	0.32425 (5)	0.12734 (5)	0.0275 (2)	
C1	0.2558 (3)	0.3471 (4)	0.3199 (3)	0.0346 (13)	
H1	0.1969	0.3397	0.3092	0.042*	
C2	0.2943 (4)	0.3585 (4)	0.3993 (3)	0.0416 (15)	
H2	0.2625	0.3604	0.4426	0.050*	
C3	0.3804 (4)	0.3672 (4)	0.4151 (3)	0.0427 (15)	
H3	0.4087	0.3741	0.4697	0.051*	
C4	0.4244 (3)	0.3657 (4)	0.3509 (3)	0.0370 (13)	
H4	0.4835	0.3715	0.3608	0.044*	
C5	0.3820 (3)	0.3557 (3)	0.2717 (3)	0.0249 (11)	
C6	0.4226 (3)	0.3574 (3)	0.1976 (3)	0.0238 (10)	
C7	0.5077 (3)	0.3676 (3)	0.1992 (3)	0.0290 (11)	
H7	0.5443	0.3718	0.2501	0.035*	
C8	0.5394 (3)	0.3716 (3)	0.1263 (3)	0.0283 (11)	
C9	0.4837 (3)	0.3648 (3)	0.0532 (3)	0.0281 (11)	
H9	0.5033	0.3662	0.0022	0.034*	
C10	0.3995 (3)	0.3559 (3)	0.0556 (3)	0.0259 (11)	
C11	0.3337 (3)	0.3549 (4)	−0.0184 (3)	0.0300 (12)	
C12	0.3511 (3)	0.3682 (4)	−0.0969 (3)	0.0376 (14)	
H12	0.4070	0.3751	−0.1059	0.045*	
C13	0.2859 (4)	0.3713 (4)	−0.1616 (4)	0.0439 (15)	
H13	0.2965	0.3822	−0.2154	0.053*	
C14	0.2048 (4)	0.3586 (4)	−0.1479 (3)	0.0408 (14)	
H14	0.1592	0.3599	−0.1918	0.049*	
C15	0.1922 (3)	0.3440 (4)	−0.0678 (3)	0.0359 (13)	
H15	0.1370	0.3346	−0.0577	0.043*	
C16	0.7731 (4)	0.3864 (5)	0.1888 (4)	0.0526 (18)	
H16	0.8130	0.3756	0.2364	0.063*	
C17	0.6904 (4)	0.3686 (5)	0.1921 (4)	0.0511 (18)	
H17	0.6751	0.3452	0.2406	0.061*	
C18	0.6299 (3)	0.3849 (4)	0.1250 (3)	0.0313 (12)	
C19	0.6575 (4)	0.4209 (5)	0.0581 (4)	0.0488 (16)	
H19	0.6187	0.4355	0.0106	0.059*	
C20	0.7424 (4)	0.4359 (5)	0.0600 (4)	0.0577 (19)	
H20	0.7598	0.4609	0.0131	0.069*	
N1	0.2981 (3)	0.3460 (3)	0.2567 (3)	0.0279 (9)	
N2	0.3699 (3)	0.3521 (3)	0.1267 (3)	0.0267 (9)	
N3	0.2550 (3)	0.3426 (3)	−0.0048 (3)	0.0290 (10)	
N4	0.8001 (3)	0.4173 (4)	0.1237 (3)	0.0484 (14)	
C21	0.3152 (3)	0.1327 (4)	0.1204 (8)	0.0301 (13)	0.85
H21	0.3661	0.1641	0.1243	0.036*	0.85
C22	0.3177 (4)	0.0437 (4)	0.1137 (8)	0.0398 (16)	0.85
H22	0.3689	0.0141	0.1125	0.048*	0.85
C23	0.2438 (4)	−0.0015 (5)	0.1086 (8)	0.0507 (19)	0.85
H23	0.2433	−0.0632	0.1038	0.061*	0.85
C24	0.1706 (4)	0.0433 (4)	0.1104 (8)	0.0450 (17)	0.85
H24	0.1194	0.0126	0.1073	0.054*	0.85
C25	0.1721 (3)	0.1329 (4)	0.1167 (6)	0.0295 (13)	0.85
C26	0.0960 (3)	0.1864 (4)	0.1156 (6)	0.0311 (14)	0.85
C27	0.0159 (4)	0.1533 (4)	0.1074 (4)	0.0333 (17)	0.85
H27	0.0070	0.0921	0.1069	0.040*	0.85
C29	−0.0365 (3)	0.2992 (5)	0.1051 (4)	0.0332 (18)	0.85
H29	−0.0815	0.3393	0.1004	0.040*	0.85
C30	0.0456 (3)	0.3287 (4)	0.1172 (6)	0.0295 (14)	0.85
C31	0.0684 (3)	0.4218 (4)	0.1253 (7)	0.0288 (13)	0.85
C32	0.0107 (4)	0.4877 (4)	0.1306 (8)	0.037 (2)	0.85
H32	−0.0469	0.4745	0.1274	0.045*	0.85
C33	0.0385 (4)	0.5721 (5)	0.1407 (4)	0.039 (2)	0.85
H33	0.0001	0.6181	0.1442	0.047*	0.85
C34	0.1223 (4)	0.5897 (4)	0.1456 (10)	0.039 (2)	0.85
H34	0.1424	0.6478	0.1524	0.047*	0.85
C35	0.1759 (4)	0.5220 (4)	0.1405 (15)	0.0365 (15)	0.85
H35	0.2337	0.5344	0.1440	0.044*	0.85
N5	0.2449 (3)	0.1775 (3)	0.1218 (7)	0.0268 (10)	0.85
N6	0.1094 (3)	0.2730 (3)	0.1208 (6)	0.0267 (10)	0.85
N7	0.1508 (3)	0.4387 (3)	0.1308 (9)	0.0282 (16)	0.85
C28	−0.0515 (4)	0.2103 (5)	0.0998 (4)	0.0295 (15)	0.85
C36	−0.2505 (6)	0.0891 (6)	0.1153 (6)	0.055 (2)	0.85
H36	−0.2682	0.0424	0.1460	0.066*	0.85
C37	−0.1654 (4)	0.1106 (5)	0.1289 (5)	0.0437 (17)	0.85
H37	−0.1266	0.0786	0.1667	0.052*	0.85
C38	−0.1400 (4)	0.1792 (5)	0.0859 (4)	0.0362 (15)	0.85
C39	−0.1991 (4)	0.2225 (5)	0.0295 (5)	0.0427 (17)	0.85
H39	−0.1833	0.2700	−0.0015	0.051*	0.85
C40	−0.2812 (4)	0.1948 (5)	0.0196 (5)	0.0455 (18)	0.85
H40	−0.3209	0.2237	−0.0200	0.055*	0.85
N8	−0.3080 (4)	0.1298 (4)	0.0623 (4)	0.0504 (16)	0.85
C121	0.3340 (10)	0.1304 (9)	0.125 (5)	0.0301 (13)	0.15
H121	0.3835	0.1642	0.1291	0.036*	0.15
C122	0.3405 (11)	0.0416 (10)	0.119 (5)	0.0398 (16)	0.15
H122	0.3932	0.0143	0.1208	0.048*	0.15
C123	0.2683 (11)	−0.0068 (9)	0.112 (5)	0.0507 (19)	0.15
H123	0.2702	−0.0682	0.1053	0.061*	0.15
C124	0.1933 (11)	0.0343 (8)	0.114 (5)	0.0450 (17)	0.15
H124	0.1435	0.0012	0.1113	0.054*	0.15
C125	0.1911 (8)	0.1238 (7)	0.122 (3)	0.0295 (13)	0.15
C126	0.1131 (8)	0.1734 (6)	0.123 (3)	0.0311 (14)	0.15
C127	0.0362 (10)	0.1360 (7)	0.126 (3)	0.0333 (17)	0.15
H127	0.0326	0.0743	0.1275	0.040*	0.15
C129	−0.0227 (9)	0.2790 (9)	0.126 (3)	0.0332 (18)	0.15
H129	−0.0685	0.3174	0.1266	0.040*	0.15
C130	0.0565 (7)	0.3130 (7)	0.125 (3)	0.0295 (14)	0.15
C131	0.0750 (7)	0.4073 (7)	0.130 (4)	0.0288 (13)	0.15
C132	0.0134 (8)	0.4711 (7)	0.119 (5)	0.037 (2)	0.15
H132	−0.0439	0.4556	0.1131	0.045*	0.15
C133	0.0370 (10)	0.5570 (8)	0.119 (3)	0.039 (2)	0.15
H133	−0.0041	0.6015	0.1084	0.047*	0.15
C134	0.1204 (10)	0.5783 (7)	0.133 (7)	0.039 (2)	0.15
H134	0.1376	0.6375	0.1362	0.047*	0.15
C135	0.1779 (9)	0.5125 (8)	0.143 (9)	0.0365 (15)	0.15
H135	0.2355	0.5274	0.1528	0.044*	0.15
N105	0.2616 (8)	0.1719 (7)	0.125 (4)	0.0268 (10)	0.15
N106	0.1221 (7)	0.2607 (6)	0.124 (3)	0.0267 (10)	0.15
N107	0.1569 (7)	0.4278 (7)	0.139 (6)	0.0282 (16)	0.15
C128	−0.036 (3)	0.185 (3)	0.127 (3)	0.029 (3)*	0.15
C136	−0.2565 (12)	0.1536 (15)	0.1642 (14)	0.029 (3)*	0.15
H136	−0.2954	0.1803	0.1935	0.035*	0.15
C137	−0.1765 (13)	0.1876 (13)	0.1695 (14)	0.029 (3)*	0.15
H137	−0.1607	0.2374	0.2023	0.035*	0.15
C138	−0.1195 (10)	0.1486 (15)	0.1266 (15)	0.029 (3)*	0.15
C139	−0.1425 (12)	0.0756 (14)	0.0785 (14)	0.029 (3)*	0.15
H139	−0.1036	0.0490	0.0492	0.035*	0.15
C140	−0.2226 (13)	0.0417 (13)	0.0732 (14)	0.029 (3)*	0.15
H140	−0.2384	−0.0082	0.0403	0.035*	0.15
N108	−0.2796 (11)	0.0807 (16)	0.1160 (16)	0.039 (13)*	0.15
P1A	0.42327 (11)	0.62842 (12)	0.19001 (12)	0.0535 (5)	0.6667
F1A	0.3907 (5)	0.5541 (6)	0.2411 (5)	0.0670 (5)*	0.6667
F2A	0.3333 (4)	0.6356 (5)	0.1268 (4)	0.0670 (5)*	0.6667
F3A	0.4501 (4)	0.7046 (4)	0.1273 (4)	0.0670 (5)*	0.6667
F4A	0.5117 (4)	0.6332 (5)	0.2411 (4)	0.0670 (5)*	0.6667
F5A	0.4558 (4)	0.5636 (5)	0.1279 (5)	0.0670 (5)*	0.6667
F6A	0.3903 (4)	0.7023 (5)	0.2432 (4)	0.0670 (5)*	0.6667
P1B	0.42327 (11)	0.62842 (12)	0.19001 (12)	0.0535 (5)	0.3333
F1B	0.3743 (9)	0.5529 (11)	0.2303 (10)	0.0670 (5)*	0.3333
F2B	0.3353 (7)	0.6633 (9)	0.1540 (8)	0.0670 (5)*	0.3333
F3B	0.4753 (8)	0.7007 (8)	0.1577 (8)	0.0670 (5)*	0.3333
F4B	0.5110 (7)	0.5810 (9)	0.2381 (8)	0.0670 (5)*	0.3333
F5B	0.4312 (8)	0.5591 (9)	0.1211 (9)	0.0670 (5)*	0.3333
F6B	0.4290 (9)	0.6817 (9)	0.2777 (9)	0.0670 (5)*	0.3333
P2A	0.01085 (12)	0.34258 (13)	0.37053 (11)	0.0515 (5)	0.6667
F7A	−0.0799 (4)	0.3052 (5)	0.3288 (4)	0.0670 (5)*	0.6667
F8A	0.0523 (6)	0.2825 (6)	0.3097 (6)	0.0670 (5)*	0.6667
F9A	0.1001 (4)	0.3682 (4)	0.4138 (4)	0.0670 (5)*	0.6667
F10A	−0.0116 (4)	0.4151 (5)	0.4360 (4)	0.0670 (5)*	0.6667
F11A	−0.0065 (4)	0.4169 (4)	0.3052 (4)	0.0670 (5)*	0.6667
F12A	0.0152 (4)	0.2618 (5)	0.4337 (5)	0.0670 (5)*	0.6667
P2B	0.01085 (12)	0.34258 (13)	0.37053 (11)	0.0515 (5)	0.3333
F7B	−0.0657 (7)	0.3666 (9)	0.3129 (7)	0.0670 (5)*	0.3333
F8B	0.0417 (11)	0.2716 (10)	0.3099 (11)	0.0670 (5)*	0.3333
F9B	0.1034 (7)	0.3303 (9)	0.4304 (8)	0.0670 (5)*	0.3333
F10B	−0.0470 (9)	0.3850 (9)	0.4251 (9)	0.0670 (5)*	0.3333
F11B	0.0566 (8)	0.4202 (8)	0.3231 (8)	0.0670 (5)*	0.3333
F12B	−0.0136 (8)	0.2662 (9)	0.4254 (9)	0.0670 (5)*	0.3333
O1W	0.5185 (7)	0.3890 (7)	0.5830 (7)	0.0411 (19)*	0.4
O2W	0.5228 (8)	0.4277 (8)	0.5626 (8)	0.0411 (19)*	0.35
O3W	0.5531 (17)	0.4242 (18)	0.5175 (17)	0.0411 (19)*	0.15
O4W	0.637 (3)	0.350 (3)	0.392 (3)	0.0411 (19)*	0.1

### (1) Bis[4'-(pyridin-4-yl)-2,2':6',2''-terpyridine]manganese(II) bis(hexafluoridophosphate) monohydrate Atomic displacement parameters (Å^2^)

**Table:**

	*U*^11^	*U*^22^	*U*^33^	*U*^12^	*U*^13^	*U*^23^
Mn1	0.0196 (4)	0.0389 (5)	0.0244 (4)	−0.0075 (3)	0.0051 (3)	−0.0017 (3)
C1	0.030 (3)	0.046 (3)	0.029 (3)	−0.003 (2)	0.009 (2)	0.000 (2)
C2	0.036 (3)	0.072 (4)	0.018 (3)	0.001 (3)	0.009 (2)	0.000 (3)
C3	0.037 (3)	0.070 (4)	0.020 (3)	0.003 (3)	0.003 (2)	−0.007 (3)
C4	0.025 (3)	0.057 (4)	0.028 (3)	−0.002 (3)	0.002 (2)	0.002 (3)
C5	0.022 (2)	0.027 (3)	0.025 (3)	−0.001 (2)	0.002 (2)	0.000 (2)
C6	0.022 (2)	0.028 (3)	0.022 (2)	−0.002 (2)	0.004 (2)	0.000 (2)
C7	0.022 (2)	0.038 (3)	0.026 (3)	0.000 (2)	0.000 (2)	0.002 (2)
C8	0.020 (2)	0.033 (3)	0.032 (3)	0.002 (2)	0.004 (2)	0.001 (2)
C9	0.025 (3)	0.035 (3)	0.026 (3)	−0.006 (2)	0.008 (2)	0.001 (2)
C10	0.023 (2)	0.031 (3)	0.023 (3)	−0.002 (2)	0.004 (2)	−0.003 (2)
C11	0.023 (3)	0.044 (3)	0.022 (3)	−0.006 (2)	0.002 (2)	−0.002 (2)
C12	0.026 (3)	0.060 (4)	0.027 (3)	−0.012 (3)	0.008 (2)	−0.004 (3)
C13	0.040 (3)	0.066 (4)	0.026 (3)	−0.008 (3)	0.006 (3)	−0.002 (3)
C14	0.030 (3)	0.064 (4)	0.027 (3)	−0.004 (3)	0.000 (2)	−0.002 (3)
C15	0.028 (3)	0.054 (4)	0.026 (3)	−0.012 (3)	0.004 (2)	0.002 (3)
C16	0.023 (3)	0.086 (5)	0.047 (4)	0.000 (3)	0.001 (3)	0.009 (4)
C17	0.022 (3)	0.088 (5)	0.043 (4)	−0.003 (3)	0.002 (3)	0.011 (3)
C18	0.020 (3)	0.036 (3)	0.039 (3)	0.004 (2)	0.007 (2)	0.003 (2)
C19	0.028 (3)	0.074 (5)	0.045 (4)	−0.002 (3)	0.008 (3)	0.004 (3)
C20	0.036 (4)	0.087 (5)	0.054 (4)	−0.014 (4)	0.020 (3)	−0.002 (4)
N1	0.024 (2)	0.033 (2)	0.027 (2)	−0.0025 (18)	0.0052 (18)	−0.0021 (19)
N2	0.021 (2)	0.034 (2)	0.026 (2)	−0.0023 (18)	0.0061 (17)	0.0006 (18)
N3	0.021 (2)	0.040 (3)	0.025 (2)	−0.0082 (19)	0.0018 (17)	−0.0023 (19)
N4	0.022 (2)	0.069 (4)	0.054 (3)	0.000 (2)	0.005 (2)	0.000 (3)
C21	0.023 (3)	0.042 (3)	0.024 (3)	0.000 (3)	−0.001 (4)	0.006 (2)
C22	0.033 (4)	0.044 (4)	0.041 (4)	0.005 (3)	0.002 (5)	0.006 (3)
C23	0.041 (5)	0.036 (4)	0.071 (5)	−0.003 (3)	−0.002 (5)	0.004 (3)
C24	0.027 (4)	0.050 (4)	0.056 (4)	−0.007 (3)	0.002 (5)	0.002 (4)
C25	0.022 (3)	0.038 (3)	0.029 (3)	−0.010 (2)	0.002 (3)	0.000 (3)
C26	0.024 (3)	0.045 (4)	0.024 (3)	−0.012 (3)	0.004 (3)	−0.002 (3)
C27	0.026 (3)	0.043 (4)	0.032 (5)	−0.009 (3)	0.007 (3)	−0.001 (3)
C29	0.019 (3)	0.048 (4)	0.034 (5)	−0.004 (3)	0.006 (3)	−0.008 (3)
C30	0.019 (3)	0.042 (3)	0.028 (3)	−0.006 (2)	0.007 (3)	−0.005 (3)
C31	0.018 (2)	0.045 (3)	0.023 (3)	−0.002 (2)	0.004 (2)	−0.002 (3)
C32	0.022 (3)	0.047 (4)	0.043 (5)	−0.005 (3)	0.009 (3)	−0.012 (4)
C33	0.029 (3)	0.050 (4)	0.038 (5)	0.002 (3)	0.005 (3)	−0.012 (3)
C34	0.041 (3)	0.034 (3)	0.042 (7)	−0.009 (3)	0.005 (3)	−0.010 (4)
C35	0.027 (3)	0.045 (4)	0.040 (4)	−0.005 (3)	0.011 (3)	−0.002 (4)
N5	0.024 (3)	0.035 (3)	0.022 (2)	−0.003 (2)	0.004 (3)	−0.001 (2)
N6	0.018 (2)	0.036 (3)	0.027 (2)	−0.005 (2)	0.006 (2)	−0.002 (2)
N7	0.018 (2)	0.039 (3)	0.028 (5)	−0.0029 (19)	0.005 (2)	−0.002 (3)
C28	0.023 (3)	0.038 (4)	0.028 (4)	−0.007 (3)	0.007 (3)	0.001 (3)
C36	0.040 (5)	0.064 (6)	0.061 (6)	−0.015 (4)	0.012 (4)	0.012 (4)
C37	0.025 (3)	0.052 (4)	0.054 (4)	−0.010 (3)	0.004 (3)	0.005 (4)
C38	0.024 (3)	0.047 (4)	0.038 (4)	−0.010 (3)	0.006 (3)	−0.007 (3)
C39	0.031 (4)	0.052 (4)	0.045 (4)	−0.010 (3)	0.006 (3)	−0.006 (3)
C40	0.026 (3)	0.058 (5)	0.050 (4)	−0.003 (3)	−0.001 (3)	−0.014 (4)
N8	0.027 (3)	0.057 (4)	0.067 (4)	−0.011 (3)	0.008 (3)	−0.004 (4)
C121	0.023 (3)	0.042 (3)	0.024 (3)	0.000 (3)	−0.001 (4)	0.006 (2)
C122	0.033 (4)	0.044 (4)	0.041 (4)	0.005 (3)	0.002 (5)	0.006 (3)
C123	0.041 (5)	0.036 (4)	0.071 (5)	−0.003 (3)	−0.002 (5)	0.004 (3)
C124	0.027 (4)	0.050 (4)	0.056 (4)	−0.007 (3)	0.002 (5)	0.002 (4)
C125	0.022 (3)	0.038 (3)	0.029 (3)	−0.010 (2)	0.002 (3)	0.000 (3)
C126	0.024 (3)	0.045 (4)	0.024 (3)	−0.012 (3)	0.004 (3)	−0.002 (3)
C127	0.026 (3)	0.043 (4)	0.032 (5)	−0.009 (3)	0.007 (3)	−0.001 (3)
C129	0.019 (3)	0.048 (4)	0.034 (5)	−0.004 (3)	0.006 (3)	−0.008 (3)
C130	0.019 (3)	0.042 (3)	0.028 (3)	−0.006 (2)	0.007 (3)	−0.005 (3)
C131	0.018 (2)	0.045 (3)	0.023 (3)	−0.002 (2)	0.004 (2)	−0.002 (3)
C132	0.022 (3)	0.047 (4)	0.043 (5)	−0.005 (3)	0.009 (3)	−0.012 (4)
C133	0.029 (3)	0.050 (4)	0.038 (5)	0.002 (3)	0.005 (3)	−0.012 (3)
C134	0.041 (3)	0.034 (3)	0.042 (7)	−0.009 (3)	0.005 (3)	−0.010 (4)
C135	0.027 (3)	0.045 (4)	0.040 (4)	−0.005 (3)	0.011 (3)	−0.002 (4)
N105	0.024 (3)	0.035 (3)	0.022 (2)	−0.003 (2)	0.004 (3)	−0.001 (2)
N106	0.018 (2)	0.036 (3)	0.027 (2)	−0.005 (2)	0.006 (2)	−0.002 (2)
N107	0.018 (2)	0.039 (3)	0.028 (5)	−0.0029 (19)	0.005 (2)	−0.002 (3)
P1A	0.0462 (10)	0.0529 (11)	0.0672 (12)	−0.0157 (8)	0.0263 (9)	−0.0193 (9)
P1B	0.0462 (10)	0.0529 (11)	0.0672 (12)	−0.0157 (8)	0.0263 (9)	−0.0193 (9)
P2A	0.0512 (10)	0.0674 (12)	0.0375 (9)	0.0224 (9)	0.0124 (8)	0.0119 (8)
P2B	0.0512 (10)	0.0674 (12)	0.0375 (9)	0.0224 (9)	0.0124 (8)	0.0119 (8)

### (1) Bis[4'-(pyridin-4-yl)-2,2':6',2''-terpyridine]manganese(II) bis(hexafluoridophosphate) monohydrate Geometric parameters (Å, º)

**Table:**

Mn1—N1	2.231 (4)	C28—C38	1.496 (9)
Mn1—N2	2.193 (4)	C36—N8	1.328 (11)
Mn1—N3	2.268 (4)	C36—C37	1.402 (12)
Mn1—N5	2.259 (5)	C36—H36	0.9500
Mn1—N6	2.212 (4)	C37—C38	1.373 (10)
Mn1—N7	2.260 (5)	C37—H37	0.9500
Mn1—N105	2.373 (11)	C38—C39	1.394 (10)
Mn1—N106	2.107 (10)	C39—C40	1.384 (9)
Mn1—N107	2.090 (16)	C39—H39	0.9500
C1—N1	1.343 (7)	C40—N8	1.336 (10)
C1—C2	1.371 (8)	C40—H40	0.9500
C1—H1	0.9500	C121—N105	1.336 (7)
C2—C3	1.387 (8)	C121—C122	1.372 (8)
C2—H2	0.9500	C121—H121	0.9500
C3—C4	1.374 (8)	C122—C123	1.377 (9)
C3—H3	0.9500	C122—H122	0.9500
C4—C5	1.388 (7)	C123—C124	1.377 (9)
C4—H4	0.9500	C123—H123	0.9500
C5—N1	1.352 (6)	C124—C125	1.380 (9)
C5—C6	1.484 (7)	C124—H124	0.9500
C6—N2	1.338 (6)	C125—N105	1.356 (7)
C6—C7	1.388 (7)	C125—C126	1.481 (8)
C7—C8	1.388 (7)	C126—N106	1.348 (7)
C7—H7	0.9500	C126—C127	1.382 (7)
C8—C9	1.393 (7)	C127—C128	1.40 (5)
C8—C18	1.487 (7)	C127—H127	0.9500
C9—C10	1.380 (7)	C129—C130	1.391 (7)
C9—H9	0.9500	C129—C128	1.47 (5)
C10—N2	1.343 (6)	C129—H129	0.9500
C10—C11	1.489 (7)	C130—N106	1.336 (7)
C11—N3	1.346 (6)	C130—C131	1.478 (8)
C11—C12	1.389 (7)	C131—N107	1.351 (6)
C12—C13	1.378 (8)	C131—C132	1.391 (8)
C12—H12	0.9500	C132—C133	1.374 (9)
C13—C14	1.385 (8)	C132—H132	0.9500
C13—H13	0.9500	C133—C134	1.375 (8)
C14—C15	1.393 (8)	C133—H133	0.9500
C14—H14	0.9500	C134—C135	1.368 (9)
C15—N3	1.334 (7)	C134—H134	0.9500
C15—H15	0.9500	C135—N107	1.343 (7)
C16—N4	1.315 (8)	C135—H135	0.9500
C16—C17	1.381 (8)	C128—C138	1.46 (5)
C16—H16	0.9500	C136—C137	1.3900
C17—C18	1.380 (8)	C136—N108	1.3900
C17—H17	0.9500	C136—H136	0.9500
C18—C19	1.376 (8)	C137—C138	1.3900
C19—C20	1.393 (8)	C137—H137	0.9500
C19—H19	0.9500	C138—C139	1.3900
C20—N4	1.322 (9)	C139—C140	1.3900
C20—H20	0.9500	C139—H139	0.9500
C21—N5	1.336 (7)	C140—N108	1.3900
C21—C22	1.372 (8)	C140—H140	0.9500
C21—H21	0.9500	P1A—F4A	1.544 (6)
C22—C23	1.377 (9)	P1A—F1A	1.564 (7)
C22—H22	0.9500	P1A—F5A	1.582 (7)
C23—C24	1.377 (9)	P1A—F6A	1.583 (7)
C23—H23	0.9500	P1A—F2A	1.656 (7)
C24—C25	1.380 (9)	P1A—F3A	1.669 (7)
C24—H24	0.9500	P1B—F3B	1.542 (11)
C25—N5	1.356 (6)	P1B—F2B	1.550 (11)
C25—C26	1.481 (8)	P1B—F5B	1.581 (11)
C26—N6	1.348 (7)	P1B—F1B	1.611 (11)
C26—C27	1.382 (7)	P1B—F6B	1.656 (14)
C27—C28	1.391 (10)	P1B—F4B	1.679 (11)
C27—H27	0.9500	P2A—F9A	1.558 (7)
C29—C28	1.386 (10)	P2A—F11A	1.565 (6)
C29—C30	1.391 (7)	P2A—F8A	1.593 (7)
C29—H29	0.9500	P2A—F12A	1.616 (7)
C30—N6	1.336 (7)	P2A—F7A	1.627 (7)
C30—C31	1.477 (8)	P2A—F10A	1.636 (7)
C31—N7	1.351 (6)	P2B—F7B	1.484 (11)
C31—C32	1.391 (8)	P2B—F10B	1.549 (14)
C32—C33	1.374 (9)	P2B—F12B	1.573 (11)
C32—H32	0.9500	P2B—F8B	1.615 (12)
C33—C34	1.375 (8)	P2B—F11B	1.668 (11)
C33—H33	0.9500	P2B—F9B	1.670 (11)
C34—C35	1.368 (9)	O1W—O2W	0.693 (13)
C34—H34	0.9500	O1W—O3W	1.41 (3)
C35—N7	1.343 (7)	O2W—O3W	0.96 (3)
C35—H35	0.9500		
			
N1—Mn1—N2	72.37 (15)	C29—C28—C38	118.6 (6)
N1—Mn1—N3	143.36 (15)	C27—C28—C38	122.3 (6)
N1—Mn1—N5	99.8 (3)	N8—C36—C37	124.3 (9)
N1—Mn1—N6	111.3 (3)	N8—C36—H36	117.9
N1—Mn1—N7	92.2 (4)	C37—C36—H36	117.9
N2—Mn1—N3	72.21 (15)	C38—C37—C36	118.0 (7)
N2—Mn1—N5	97.87 (16)	C38—C37—H37	121.0
N2—Mn1—N6	169.95 (18)	C36—C37—H37	121.0
N2—Mn1—N7	117.68 (17)	C37—C38—C39	118.7 (6)
N3—Mn1—N5	94.0 (3)	C37—C38—C28	121.9 (7)
N3—Mn1—N6	105.2 (3)	C39—C38—C28	119.4 (6)
N3—Mn1—N7	96.0 (4)	C40—C39—C38	118.5 (7)
N5—Mn1—N6	72.45 (17)	C40—C39—H39	120.7
N5—Mn1—N7	144.44 (17)	C38—C39—H39	120.7
N6—Mn1—N7	71.99 (17)	N8—C40—C39	123.9 (7)
N107—Mn1—N106	77.6 (5)	N8—C40—H40	118.1
N107—Mn1—N2	118.8 (4)	C39—C40—H40	118.1
N106—Mn1—N2	163.6 (3)	C36—N8—C40	116.5 (7)
N107—Mn1—N1	89 (2)	N105—C121—C122	123.4 (6)
N106—Mn1—N1	110.1 (14)	N105—C121—H121	118.3
N107—Mn1—N3	100 (3)	C122—C121—H121	118.3
N106—Mn1—N3	106.5 (14)	C121—C122—C123	118.0 (6)
N107—Mn1—N105	149.2 (10)	C121—C122—H122	121.0
N106—Mn1—N105	72.0 (3)	C123—C122—H122	121.0
N2—Mn1—N105	91.6 (3)	C122—C123—C124	119.6 (7)
N1—Mn1—N105	96.8 (16)	C122—C123—H123	120.2
N3—Mn1—N105	93.3 (17)	C124—C123—H123	120.2
N1—C1—C2	122.4 (5)	C123—C124—C125	119.6 (6)
N1—C1—H1	118.8	C123—C124—H124	120.2
C2—C1—H1	118.8	C125—C124—H124	120.2
C1—C2—C3	118.8 (5)	N105—C125—C124	120.7 (6)
C1—C2—H2	120.6	N105—C125—C126	116.0 (5)
C3—C2—H2	120.6	C124—C125—C126	123.2 (5)
C4—C3—C2	119.2 (5)	N106—C126—C127	120.5 (6)
C4—C3—H3	120.4	N106—C126—C125	114.9 (5)
C2—C3—H3	120.4	C127—C126—C125	124.5 (6)
C3—C4—C5	119.5 (5)	C126—C127—C128	123 (2)
C3—C4—H4	120.3	C126—C127—H127	118.4
C5—C4—H4	120.3	C128—C127—H127	118.4
N1—C5—C4	121.0 (5)	C130—C129—C128	120.8 (19)
N1—C5—C6	114.9 (4)	C130—C129—H129	119.6
C4—C5—C6	124.1 (4)	C128—C129—H129	119.6
N2—C6—C7	121.0 (4)	N106—C130—C129	121.0 (6)
N2—C6—C5	114.7 (4)	N106—C130—C131	115.7 (4)
C7—C6—C5	124.2 (4)	C129—C130—C131	123.2 (6)
C8—C7—C6	119.9 (5)	N107—C131—C132	121.7 (5)
C8—C7—H7	120.1	N107—C131—C130	115.0 (5)
C6—C7—H7	120.1	C132—C131—C130	123.2 (5)
C7—C8—C9	118.2 (5)	C133—C132—C131	118.8 (5)
C7—C8—C18	121.8 (5)	C133—C132—H132	120.6
C9—C8—C18	120.0 (5)	C131—C132—H132	120.6
C10—C9—C8	119.3 (5)	C132—C133—C134	119.7 (6)
C10—C9—H9	120.4	C132—C133—H133	120.2
C8—C9—H9	120.4	C134—C133—H133	120.2
N2—C10—C9	121.8 (5)	C135—C134—C133	118.6 (6)
N2—C10—C11	114.1 (4)	C135—C134—H134	120.7
C9—C10—C11	124.0 (5)	C133—C134—H134	120.7
N3—C11—C12	121.4 (5)	N107—C135—C134	123.1 (6)
N3—C11—C10	115.9 (4)	N107—C135—H135	118.5
C12—C11—C10	122.7 (5)	C134—C135—H135	118.5
C13—C12—C11	118.9 (5)	C121—N105—C125	118.6 (5)
C13—C12—H12	120.6	C121—N105—Mn1	128.0 (5)
C11—C12—H12	120.6	C125—N105—Mn1	113.4 (4)
C12—C13—C14	119.9 (5)	C130—N106—C126	120.8 (5)
C12—C13—H13	120.0	C130—N106—Mn1	115.5 (5)
C14—C13—H13	120.0	C126—N106—Mn1	123.7 (5)
C13—C14—C15	118.0 (5)	C135—N107—C131	118.0 (5)
C13—C14—H14	121.0	C135—N107—Mn1	125.5 (13)
C15—C14—H14	121.0	C131—N107—Mn1	115.6 (6)
N3—C15—C14	122.3 (5)	C127—C128—C138	125 (3)
N3—C15—H15	118.8	C127—C128—C129	114 (3)
C14—C15—H15	118.8	C138—C128—C129	121 (3)
N4—C16—C17	124.0 (6)	C137—C136—N108	120.0
N4—C16—H16	118.0	C137—C136—H136	120.0
C17—C16—H16	118.0	N108—C136—H136	120.0
C18—C17—C16	120.1 (6)	C136—C137—C138	120.0
C18—C17—H17	119.9	C136—C137—H137	120.0
C16—C17—H17	119.9	C138—C137—H137	120.0
C19—C18—C17	115.9 (5)	C139—C138—C137	120.0
C19—C18—C8	121.3 (5)	C139—C138—C128	118 (2)
C17—C18—C8	122.5 (5)	C137—C138—C128	122 (2)
C18—C19—C20	120.0 (6)	C140—C139—C138	120.0
C18—C19—H19	120.0	C140—C139—H139	120.0
C20—C19—H19	120.0	C138—C139—H139	120.0
N4—C20—C19	123.5 (6)	C139—C140—N108	120.0
N4—C20—H20	118.3	C139—C140—H140	120.0
C19—C20—H20	118.3	N108—C140—H140	120.0
C1—N1—C5	119.0 (4)	C140—N108—C136	120.0
C1—N1—Mn1	123.2 (4)	F4A—P1A—F1A	96.0 (4)
C5—N1—Mn1	117.7 (3)	F4A—P1A—F5A	90.4 (4)
C6—N2—C10	119.9 (4)	F1A—P1A—F5A	94.1 (5)
C6—N2—Mn1	119.6 (3)	F4A—P1A—F6A	91.7 (4)
C10—N2—Mn1	120.2 (3)	F1A—P1A—F6A	92.7 (5)
C15—N3—C11	119.4 (4)	F5A—P1A—F6A	172.7 (4)
C15—N3—Mn1	123.8 (3)	F4A—P1A—F2A	171.2 (4)
C11—N3—Mn1	116.8 (3)	F1A—P1A—F2A	92.8 (4)
C16—N4—C20	116.4 (5)	F5A—P1A—F2A	89.2 (4)
N5—C21—C22	123.4 (5)	F6A—P1A—F2A	87.7 (4)
N5—C21—H21	118.3	F4A—P1A—F3A	89.6 (4)
C22—C21—H21	118.3	F1A—P1A—F3A	173.9 (4)
C21—C22—C23	118.0 (6)	F5A—P1A—F3A	83.5 (4)
C21—C22—H22	121.0	F6A—P1A—F3A	89.5 (4)
C23—C22—H22	121.0	F2A—P1A—F3A	81.5 (3)
C22—C23—C24	119.6 (6)	F3B—P1B—F2B	98.1 (7)
C22—C23—H23	120.2	F3B—P1B—F5B	96.9 (7)
C24—C23—H23	120.2	F2B—P1B—F5B	97.7 (7)
C23—C24—C25	119.7 (6)	F3B—P1B—F1B	175.5 (8)
C23—C24—H24	120.2	F2B—P1B—F1B	85.5 (7)
C25—C24—H24	120.2	F5B—P1B—F1B	85.3 (9)
N5—C25—C24	120.8 (5)	F3B—P1B—F6B	89.6 (7)
N5—C25—C26	116.0 (5)	F2B—P1B—F6B	94.7 (7)
C24—C25—C26	123.2 (5)	F5B—P1B—F6B	165.0 (8)
N6—C26—C27	120.5 (6)	F1B—P1B—F6B	87.3 (9)
N6—C26—C25	114.9 (4)	F3B—P1B—F4B	90.3 (7)
C27—C26—C25	124.5 (5)	F2B—P1B—F4B	171.3 (7)
C26—C27—C28	119.4 (6)	F5B—P1B—F4B	83.5 (7)
C26—C27—H27	120.3	F1B—P1B—F4B	86.0 (7)
C28—C27—H27	120.3	F6B—P1B—F4B	82.9 (7)
C28—C29—C30	118.9 (6)	F9A—P2A—F11A	100.5 (4)
C28—C29—H29	120.5	F9A—P2A—F8A	88.8 (4)
C30—C29—H29	120.5	F11A—P2A—F8A	91.9 (4)
N6—C30—C29	121.1 (5)	F9A—P2A—F12A	87.5 (4)
N6—C30—C31	115.7 (4)	F11A—P2A—F12A	171.9 (4)
C29—C30—C31	123.2 (5)	F8A—P2A—F12A	89.2 (4)
N7—C31—C32	121.7 (5)	F9A—P2A—F7A	173.8 (4)
N7—C31—C30	115.0 (5)	F11A—P2A—F7A	85.1 (4)
C32—C31—C30	123.2 (5)	F8A—P2A—F7A	88.5 (4)
C33—C32—C31	118.8 (5)	F12A—P2A—F7A	86.9 (4)
C33—C32—H32	120.6	F9A—P2A—F10A	79.3 (4)
C31—C32—H32	120.6	F11A—P2A—F10A	85.8 (4)
C32—C33—C34	119.7 (6)	F8A—P2A—F10A	167.3 (4)
C32—C33—H33	120.1	F12A—P2A—F10A	94.8 (4)
C34—C33—H33	120.1	F7A—P2A—F10A	103.7 (4)
C35—C34—C33	118.7 (6)	F7B—P2B—F10B	75.3 (7)
C35—C34—H34	120.7	F7B—P2B—F12B	107.1 (7)
C33—C34—H34	120.7	F10B—P2B—F12B	75.1 (8)
N7—C35—C34	123.1 (5)	F7B—P2B—F8B	94.6 (9)
N7—C35—H35	118.4	F10B—P2B—F8B	157.7 (8)
C34—C35—H35	118.4	F12B—P2B—F8B	89.4 (8)
C21—N5—C25	118.6 (5)	F7B—P2B—F11B	84.9 (7)
C21—N5—Mn1	124.3 (4)	F10B—P2B—F11B	109.5 (7)
C25—N5—Mn1	117.1 (4)	F12B—P2B—F11B	168.1 (7)
C30—N6—C26	120.8 (4)	F8B—P2B—F11B	89.0 (7)
C30—N6—Mn1	119.5 (3)	F7B—P2B—F9B	170.8 (7)
C26—N6—Mn1	119.6 (4)	F10B—P2B—F9B	105.9 (7)
C35—N7—C31	118.0 (5)	F12B—P2B—F9B	82.0 (6)
C35—N7—Mn1	124.3 (4)	F8B—P2B—F9B	87.3 (8)
C31—N7—Mn1	117.6 (4)	F11B—P2B—F9B	86.1 (7)
C29—C28—C27	119.1 (6)	O1W—O2W—O3W	116 (3)
			
N1—C1—C2—C3	−1.5 (10)	C31—C30—N6—C26	178.8 (8)
C1—C2—C3—C4	1.1 (10)	C29—C30—N6—Mn1	173.7 (7)
C2—C3—C4—C5	0.1 (10)	C31—C30—N6—Mn1	−5.2 (9)
C3—C4—C5—N1	−1.1 (9)	C27—C26—N6—C30	−0.7 (12)
C3—C4—C5—C6	177.0 (5)	C25—C26—N6—C30	177.3 (7)
N1—C5—C6—N2	2.3 (7)	C27—C26—N6—Mn1	−176.8 (7)
C4—C5—C6—N2	−175.9 (5)	C25—C26—N6—Mn1	1.3 (10)
N1—C5—C6—C7	179.3 (5)	C34—C35—N7—C31	0 (2)
C4—C5—C6—C7	1.1 (8)	C34—C35—N7—Mn1	−177.4 (15)
N2—C6—C7—C8	−0.7 (8)	C32—C31—N7—C35	−1.0 (15)
C5—C6—C7—C8	−177.5 (5)	C30—C31—N7—C35	−178.2 (12)
C6—C7—C8—C9	−0.3 (8)	C32—C31—N7—Mn1	177.0 (9)
C6—C7—C8—C18	178.3 (5)	C30—C31—N7—Mn1	−0.3 (10)
C7—C8—C9—C10	1.2 (8)	C30—C29—C28—C27	0.4 (10)
C18—C8—C9—C10	−177.5 (5)	C30—C29—C28—C38	−179.3 (7)
C8—C9—C10—N2	−1.2 (8)	C26—C27—C28—C29	−3.3 (11)
C8—C9—C10—C11	175.0 (5)	C26—C27—C28—C38	176.4 (7)
N2—C10—C11—N3	−5.8 (7)	N8—C36—C37—C38	1.3 (13)
C9—C10—C11—N3	177.8 (5)	C36—C37—C38—C39	−1.7 (11)
N2—C10—C11—C12	172.5 (5)	C36—C37—C38—C28	176.3 (7)
C9—C10—C11—C12	−3.9 (9)	C29—C28—C38—C37	−135.8 (8)
N3—C11—C12—C13	1.9 (9)	C27—C28—C38—C37	44.4 (10)
C10—C11—C12—C13	−176.3 (6)	C29—C28—C38—C39	42.1 (9)
C11—C12—C13—C14	−2.0 (10)	C27—C28—C38—C39	−137.6 (7)
C12—C13—C14—C15	0.7 (10)	C37—C38—C39—C40	0.4 (11)
C13—C14—C15—N3	0.6 (10)	C28—C38—C39—C40	−177.6 (6)
N4—C16—C17—C18	−1.4 (12)	C38—C39—C40—N8	1.6 (11)
C16—C17—C18—C19	−1.5 (10)	C37—C36—N8—C40	0.5 (13)
C16—C17—C18—C8	−176.6 (6)	C39—C40—N8—C36	−1.9 (12)
C7—C8—C18—C19	−155.2 (6)	N105—C121—C122—C123	1 (11)
C9—C8—C18—C19	23.4 (8)	C121—C122—C123—C124	−3 (8)
C7—C8—C18—C17	19.7 (9)	C122—C123—C124—C125	2 (9)
C9—C8—C18—C17	−161.7 (6)	C123—C124—C125—N105	1 (8)
C17—C18—C19—C20	2.1 (10)	C123—C124—C125—C126	179 (6)
C8—C18—C19—C20	177.3 (6)	N105—C125—C126—N106	2 (5)
C18—C19—C20—N4	0.1 (12)	C124—C125—C126—N106	−175 (5)
C2—C1—N1—C5	0.6 (8)	N105—C125—C126—C127	−175 (5)
C2—C1—N1—Mn1	178.4 (5)	C124—C125—C126—C127	7 (6)
C4—C5—N1—C1	0.8 (8)	N106—C126—C127—C128	3 (7)
C6—C5—N1—C1	−177.5 (5)	C125—C126—C127—C128	−180 (4)
C4—C5—N1—Mn1	−177.2 (4)	C128—C129—C130—N106	0 (6)
C6—C5—N1—Mn1	4.5 (6)	C128—C129—C130—C131	−176 (4)
C7—C6—N2—C10	0.7 (8)	N106—C130—C131—N107	−5 (4)
C5—C6—N2—C10	177.8 (5)	C129—C130—C131—N107	171 (5)
C7—C6—N2—Mn1	174.5 (4)	N106—C130—C131—C132	171 (5)
C5—C6—N2—Mn1	−8.3 (6)	C129—C130—C131—C132	−13 (5)
C9—C10—N2—C6	0.2 (8)	N107—C131—C132—C133	1 (7)
C11—C10—N2—C6	−176.3 (5)	C130—C131—C132—C133	−175 (6)
C9—C10—N2—Mn1	−173.6 (4)	C131—C132—C133—C134	−4 (6)
C11—C10—N2—Mn1	9.9 (6)	C132—C133—C134—C135	4 (9)
C14—C15—N3—C11	−0.7 (9)	C133—C134—C135—N107	0 (13)
C14—C15—N3—Mn1	177.6 (5)	C122—C121—N105—C125	2 (11)
C12—C11—N3—C15	−0.6 (9)	C122—C121—N105—Mn1	−177 (6)
C10—C11—N3—C15	177.7 (5)	C124—C125—N105—C121	−3 (7)
C12—C11—N3—Mn1	−179.0 (5)	C126—C125—N105—C121	179 (5)
C10—C11—N3—Mn1	−0.6 (6)	C124—C125—N105—Mn1	176 (5)
C17—C16—N4—C20	3.5 (12)	C126—C125—N105—Mn1	−2 (5)
C19—C20—N4—C16	−2.9 (11)	C129—C130—N106—C126	2 (5)
N5—C21—C22—C23	−0.4 (18)	C131—C130—N106—C126	178 (4)
C21—C22—C23—C24	0.0 (16)	C129—C130—N106—Mn1	−177 (4)
C22—C23—C24—C25	0.4 (16)	C131—C130—N106—Mn1	−1 (4)
C23—C24—C25—N5	−0.5 (15)	C127—C126—N106—C130	−3 (6)
C23—C24—C25—C26	177.4 (10)	C125—C126—N106—C130	180 (3)
N5—C25—C26—N6	−0.8 (10)	C127—C126—N106—Mn1	176 (4)
C24—C25—C26—N6	−178.7 (9)	C125—C126—N106—Mn1	−1 (5)
N5—C25—C26—C27	177.2 (9)	C134—C135—N107—C131	−3 (11)
C24—C25—C26—C27	−0.7 (12)	C134—C135—N107—Mn1	165 (9)
N6—C26—C27—C28	3.6 (12)	C132—C131—N107—C135	3 (8)
C25—C26—C27—C28	−174.3 (7)	C130—C131—N107—C135	179 (6)
C28—C29—C30—N6	2.5 (11)	C132—C131—N107—Mn1	−167 (5)
C28—C29—C30—C31	−178.7 (7)	C130—C131—N107—Mn1	9 (5)
N6—C30—C31—N7	3.5 (10)	C126—C127—C128—C138	177 (4)
C29—C30—C31—N7	−175.4 (9)	C126—C127—C128—C129	−1 (7)
N6—C30—C31—C32	−173.7 (9)	C130—C129—C128—C127	0 (6)
C29—C30—C31—C32	7.4 (13)	C130—C129—C128—C138	−179 (4)
N7—C31—C32—C33	0.9 (13)	N108—C136—C137—C138	0.0
C30—C31—C32—C33	177.9 (9)	C136—C137—C138—C139	0.0
C31—C32—C33—C34	−0.3 (13)	C136—C137—C138—C128	177 (3)
C32—C33—C34—C35	−0.2 (16)	C127—C128—C138—C139	−39 (5)
C33—C34—C35—N7	0 (2)	C129—C128—C138—C139	140 (4)
C22—C21—N5—C25	0.4 (17)	C127—C128—C138—C137	144 (4)
C22—C21—N5—Mn1	−177.3 (10)	C129—C128—C138—C137	−37 (5)
C24—C25—N5—C21	0.0 (14)	C137—C138—C139—C140	0.0
C26—C25—N5—C21	−177.9 (9)	C128—C138—C139—C140	−177 (3)
C24—C25—N5—Mn1	177.9 (8)	C138—C139—C140—N108	0.0
C26—C25—N5—Mn1	−0.1 (10)	C139—C140—N108—C136	0.0
C29—C30—N6—C26	−2.3 (12)	C137—C136—N108—C140	0.0

### (1) Bis[4'-(pyridin-4-yl)-2,2':6',2''-terpyridine]manganese(II) bis(hexafluoridophosphate) monohydrate Hydrogen-bond geometry (Å, º)

**Table:**

*D*—H···*A*	*D*—H	H···*A*	*D*···*A*	*D*—H···*A*
C12—H12···F5*A*^i^	0.95	2.50	3.422 (9)	164
C15—H15···F12*A*^ii^	0.95	2.46	3.305 (9)	149
C16—H16···F7*A*^iii^	0.95	2.38	3.289 (9)	160
C19—H19···F5*A*^i^	0.95	2.41	3.328 (10)	162
C29—H29···N4^iv^	0.95	2.35	3.270 (8)	163

Symmetry codes: (i) −*x*+1, −*y*+1, −*z*; (ii) *x*, −*y*+1/2, *z*−1/2; (iii) *x*+1, *y*, *z*; (iv) *x*−1, *y*, *z*.

### (2) Bis[4'-(pyridin-4-yl)-2,2':6',2''-terpyridine]manganese(II) bis(hexafluoridophosphate) acetone monosolvate Crystal data

**Table:**

[Mn(C_20_H_14_N_4_)_2_](PF_6_)_2_·C_3_H_6_O	*D*_x_ = 1.595 Mg m^−^^3^
*M**_r_* = 1023.66	Mo *K*α radiation, λ = 0.71073 Å
Orthorhombic, *C*222_1_	Cell parameters from 6510 reflections
*a* = 18.0996 (15) Å	θ = 2.7–25.2°
*b* = 27.470 (2) Å	µ = 0.48 mm^−^^1^
*c* = 8.5734 (6) Å	*T* = 150 K
*V* = 4262.7 (6) Å^3^	Block, yellow
*Z* = 4	0.16 × 0.08 × 0.04 mm
*F*(000) = 2076	

### (2) Bis[4'-(pyridin-4-yl)-2,2':6',2''-terpyridine]manganese(II) bis(hexafluoridophosphate) acetone monosolvate Data collection

**Table:**

Bruker APEXII CCD diffractometer	3908 independent reflections
Radiation source: sealed tube	2683 reflections with *I* > 2σ(*I*)
Graphite monochromator	*R*_int_ = 0.091
φ and ω scans	θ_max_ = 25.4°, θ_min_ = 2.3°
Absorption correction: multi-scan (SADABS; Bruker, 2007)	*h* = −21→21
*T*_min_ = 0.955, *T*_max_ = 0.981	*k* = −31→33
18921 measured reflections	*l* = −10→10

### (2) Bis[4'-(pyridin-4-yl)-2,2':6',2''-terpyridine]manganese(II) bis(hexafluoridophosphate) acetone monosolvate Refinement

**Table:**

Refinement on *F*^2^	Hydrogen site location: inferred from neighbouring sites
Least-squares matrix: full	H-atom parameters constrained
*R*[*F*^2^ > 2σ(*F*^2^)] = 0.104	*w* = 1/[σ^2^(*F*_o_^2^) + (0.0919*P*)^2^ + 48.9339*P*] where *P* = (*F*_o_^2^ + 2*F*_c_^2^)/3
*wR*(*F*^2^) = 0.270	(Δ/σ)_max_ < 0.001
*S* = 1.07	Δρ_max_ = 0.71 e Å^−^^3^
3908 reflections	Δρ_min_ = −1.05 e Å^−^^3^
292 parameters	Absolute structure: Flack *x* determined using 836 quotients [(I+)-(I-)]/[(I+)+(I-)] (Parsons *et al.* (2013)
102 restraints	Absolute structure parameter: 0.21 (2)

### (2) Bis[4'-(pyridin-4-yl)-2,2':6',2''-terpyridine]manganese(II) bis(hexafluoridophosphate) acetone monosolvate Special details

**Table:**

Geometry. All e.s.d.'s (except the e.s.d. in the dihedral angle between two l.s. planes) are estimated using the full covariance matrix. The cell e.s.d.'s are taken into account individually in the estimation of e.s.d.'s in distances, angles and torsion angles; correlations between e.s.d.'s in cell parameters are only used when they are defined by crystal symmetry. An approximate (isotropic) treatment of cell e.s.d.'s is used for estimating e.s.d.'s involving l.s. planes.

### (2) Bis[4'-(pyridin-4-yl)-2,2':6',2''-terpyridine]manganese(II) bis(hexafluoridophosphate) acetone monosolvate Fractional atomic coordinates and isotropic or equivalent isotropic displacement parameters (Å^2^)

**Table:**

	*x*	*y*	*z*	*U*_iso_*/*U*_eq_	Occ. (<1)
Mn1	0.5000	0.77635 (9)	0.7500	0.0370 (7)	
C1	0.4292 (7)	0.7832 (5)	1.0872 (14)	0.048 (3)	
H1	0.4307	0.8172	1.0665	0.058*	
C2	0.4020 (7)	0.7676 (6)	1.2295 (16)	0.054 (4)	
H2	0.3866	0.7902	1.3069	0.065*	
C3	0.3980 (7)	0.7181 (5)	1.2545 (16)	0.048 (3)	
H3	0.3784	0.7062	1.3500	0.058*	
C4	0.4219 (7)	0.6858 (5)	1.1433 (14)	0.043 (3)	
H4	0.4197	0.6517	1.1621	0.051*	
C5	0.4488 (7)	0.7032 (5)	1.0052 (13)	0.039 (3)	
C6	0.4751 (6)	0.6721 (4)	0.8756 (12)	0.032 (3)	
C7	0.4750 (7)	0.6216 (5)	0.8755 (15)	0.048 (3)	
H7	0.4573	0.6046	0.9644	0.058*	
C8	0.5000	0.5958 (6)	0.7500	0.044 (4)	
C12	0.3343 (7)	0.7696 (6)	0.6153 (14)	0.050 (4)	
H12	0.3387	0.7357	0.6366	0.060*	
C13	0.2723 (8)	0.7871 (6)	0.5405 (16)	0.056 (4)	
H13	0.2342	0.7651	0.5120	0.068*	
C14	0.2645 (8)	0.8344 (7)	0.5069 (18)	0.061 (4)	
H14	0.2217	0.8457	0.4539	0.073*	
C15	0.3211 (7)	0.8675 (6)	0.5515 (17)	0.054 (4)	
H15	0.3180	0.9012	0.5279	0.064*	
C16	0.3811 (7)	0.8483 (5)	0.6310 (14)	0.040 (3)	
C17	0.4424 (7)	0.8814 (5)	0.6884 (13)	0.039 (3)	
C18	0.4412 (7)	0.9303 (5)	0.6864 (14)	0.041 (3)	
H18	0.4002	0.9468	0.6417	0.049*	
C19	0.5000	0.9574 (6)	0.7500	0.046 (4)	
C20	0.5000	1.0098 (7)	0.7500	0.054 (5)	
C21	0.4345 (8)	1.0377 (5)	0.7816 (19)	0.058 (4)	
H21	0.3892	1.0217	0.8040	0.069*	
C22	0.4377 (9)	1.0875 (5)	0.779 (2)	0.071 (5)	
H22	0.3935	1.1051	0.7990	0.085*	
N1	0.4541 (6)	0.7520 (4)	0.9758 (11)	0.039 (3)	
N2	0.5000	0.6970 (5)	0.7500	0.035 (3)	
N4	0.5000	0.8560 (5)	0.7500	0.041 (3)	
N5	0.3898 (6)	0.8012 (4)	0.6593 (11)	0.039 (2)	
N6	0.5000	1.1129 (5)	0.7500	0.070 (5)	
C109	0.5000	0.5411 (6)	0.7500	0.049 (4)	0.5
C110	0.4556 (18)	0.5139 (11)	0.646 (4)	0.058 (6)	0.5
H110	0.4251	0.5288	0.5695	0.070*	0.5
C111	0.460 (2)	0.4652 (12)	0.663 (4)	0.061 (6)	0.5
H111	0.4270	0.4475	0.5977	0.074*	0.5
N13	0.5000	0.4386 (6)	0.7500	0.080 (6)	0.5
C209	0.5000	0.5411 (6)	0.7500	0.049 (4)	0.5
C210	0.4316 (18)	0.5177 (10)	0.765 (4)	0.058 (6)	0.5
H210	0.3863	0.5347	0.7785	0.070*	0.5
C211	0.436 (2)	0.4708 (10)	0.758 (4)	0.061 (6)	0.5
H211	0.3894	0.4545	0.7588	0.074*	0.5
N23	0.5000	0.4386 (6)	0.7500	0.080 (6)	0.5
P1	0.7697 (4)	0.8833 (3)	0.4862 (10)	0.0683 (16)*	0.4
F11	0.7203 (7)	0.8354 (4)	0.4710 (17)	0.0978 (19)*	0.4
F12	0.7479 (8)	0.8984 (5)	0.3127 (13)	0.0978 (19)*	0.4
F13	0.8190 (7)	0.9311 (4)	0.5015 (17)	0.0978 (19)*	0.4
F14	0.7915 (8)	0.8681 (5)	0.6597 (13)	0.0978 (19)*	0.4
F15	0.8393 (7)	0.8541 (5)	0.4211 (17)	0.0978 (19)*	0.4
F16	0.7000 (7)	0.9124 (5)	0.5513 (17)	0.0978 (19)*	0.4
P2	0.7698 (5)	0.8722 (3)	0.4736 (11)	0.0683 (16)*	0.6
F21	0.7250 (10)	0.8513 (7)	0.330 (2)	0.0978 (19)*	0.6
F22	0.7976 (10)	0.9225 (7)	0.422 (2)	0.0978 (19)*	0.6
F23	0.8155 (10)	0.8818 (7)	0.635 (2)	0.0978 (19)*	0.6
F24	0.7524 (10)	0.8204 (7)	0.557 (2)	0.0978 (19)*	0.6
F25	0.8441 (10)	0.8478 (7)	0.396 (2)	0.0978 (19)*	0.6
F26	0.6985 (10)	0.8939 (7)	0.548 (2)	0.0978 (19)*	0.6
O90	0.8075 (9)	0.5064 (8)	0.520 (3)	0.066 (6)*	0.5
C91	0.746 (2)	0.4862 (10)	0.754 (3)	0.126 (10)*	0.5
H91A	0.7806	0.4587	0.7596	0.188*	0.5
H91B	0.6956	0.4745	0.7697	0.188*	0.5
H91C	0.7586	0.5100	0.8350	0.188*	0.5
C90	0.7521 (10)	0.5095 (4)	0.599 (2)	0.126 (10)*	0.5
C92	0.6893 (17)	0.5377 (10)	0.534 (5)	0.126 (10)*	0.5
H92A	0.6432	0.5200	0.5533	0.188*	0.5
H92B	0.6962	0.5421	0.4218	0.188*	0.5
H92C	0.6870	0.5696	0.5853	0.188*	0.5

### (2) Bis[4'-(pyridin-4-yl)-2,2':6',2''-terpyridine]manganese(II) bis(hexafluoridophosphate) acetone monosolvate Atomic displacement parameters (Å^2^)

**Table:**

	*U*^11^	*U*^22^	*U*^33^	*U*^12^	*U*^13^	*U*^23^
Mn1	0.0534 (15)	0.0331 (13)	0.0246 (11)	0.000	0.0046 (13)	0.000
C1	0.055 (8)	0.054 (9)	0.036 (6)	−0.007 (7)	0.017 (6)	−0.010 (6)
C2	0.051 (8)	0.072 (10)	0.039 (8)	−0.006 (7)	0.007 (6)	−0.010 (8)
C3	0.052 (7)	0.066 (9)	0.028 (5)	−0.003 (6)	0.008 (6)	−0.006 (9)
C4	0.053 (8)	0.048 (8)	0.026 (6)	−0.004 (6)	0.001 (6)	0.003 (6)
C5	0.046 (7)	0.047 (8)	0.024 (6)	0.002 (6)	0.001 (5)	−0.003 (5)
C6	0.039 (7)	0.038 (7)	0.020 (5)	0.001 (5)	0.001 (5)	0.001 (5)
C7	0.059 (9)	0.052 (8)	0.034 (6)	−0.009 (6)	0.000 (6)	0.009 (6)
C8	0.055 (11)	0.032 (9)	0.044 (10)	0.000	−0.011 (12)	0.000
C12	0.036 (7)	0.082 (11)	0.033 (6)	−0.013 (7)	0.004 (6)	0.004 (7)
C13	0.040 (7)	0.083 (8)	0.046 (8)	−0.017 (8)	0.006 (6)	−0.010 (7)
C14	0.035 (7)	0.091 (9)	0.056 (9)	−0.004 (8)	0.008 (7)	0.004 (8)
C15	0.029 (7)	0.080 (11)	0.053 (8)	−0.002 (7)	−0.001 (6)	0.011 (8)
C16	0.033 (6)	0.050 (8)	0.036 (6)	−0.007 (6)	0.002 (5)	−0.003 (6)
C17	0.046 (7)	0.042 (8)	0.027 (5)	−0.006 (6)	0.004 (5)	−0.008 (5)
C18	0.039 (7)	0.046 (8)	0.039 (7)	0.005 (6)	0.004 (6)	0.002 (5)
C19	0.042 (10)	0.035 (9)	0.060 (11)	0.000	−0.003 (12)	0.000
C20	0.045 (10)	0.057 (12)	0.059 (12)	0.000	0.012 (13)	0.000
C21	0.053 (9)	0.054 (9)	0.066 (11)	0.008 (7)	0.005 (8)	0.003 (8)
C22	0.065 (10)	0.044 (8)	0.105 (16)	0.012 (7)	0.011 (10)	0.002 (9)
N1	0.057 (7)	0.039 (6)	0.022 (5)	−0.002 (5)	0.002 (5)	−0.004 (4)
N2	0.051 (8)	0.035 (7)	0.020 (6)	0.000	−0.005 (8)	0.000
N4	0.049 (8)	0.047 (8)	0.027 (7)	0.000	−0.010 (8)	0.000
N5	0.047 (6)	0.038 (6)	0.030 (5)	−0.007 (5)	0.007 (5)	0.001 (4)
N6	0.061 (11)	0.032 (9)	0.116 (15)	0.000	0.007 (14)	0.000
C109	0.075 (13)	0.029 (9)	0.042 (9)	0.000	−0.003 (13)	0.000
C110	0.069 (15)	0.046 (12)	0.060 (14)	0.004 (10)	−0.017 (13)	−0.012 (12)
C111	0.086 (18)	0.046 (12)	0.052 (16)	−0.001 (11)	0.002 (14)	−0.005 (14)
N13	0.132 (18)	0.034 (10)	0.075 (12)	0.000	−0.027 (16)	0.000
C209	0.075 (13)	0.029 (9)	0.042 (9)	0.000	−0.003 (13)	0.000
C210	0.069 (15)	0.046 (12)	0.060 (14)	0.004 (10)	−0.017 (13)	−0.012 (12)
C211	0.086 (18)	0.046 (12)	0.052 (16)	−0.001 (11)	0.002 (14)	−0.005 (14)
N23	0.132 (18)	0.034 (10)	0.075 (12)	0.000	−0.027 (16)	0.000

### (2) Bis[4'-(pyridin-4-yl)-2,2':6',2''-terpyridine]manganese(II) bis(hexafluoridophosphate) acetone monosolvate Geometric parameters (Å, º)

**Table:**

Mn1—N1	2.210 (10)	C21—C22	1.37 (2)
Mn1—N2	2.180 (13)	C21—H21	0.9500
Mn1—N4	2.187 (14)	C22—N6	1.351 (18)
Mn1—N5	2.247 (11)	C22—H22	0.9500
Mn1—N1^i^	2.210 (10)	N2—C6^i^	1.353 (13)
Mn1—N5^i^	2.247 (11)	N4—C17^i^	1.361 (15)
C1—N1	1.358 (16)	N6—C22^i^	1.351 (18)
C1—C2	1.384 (18)	C109—C110	1.41 (3)
C1—H1	0.9500	C109—C110^i^	1.41 (3)
C2—C3	1.379 (19)	C110—C111	1.35 (4)
C2—H2	0.9500	C110—H110	0.9500
C3—C4	1.372 (18)	C111—N13	1.27 (3)
C3—H3	0.9500	C111—H111	0.9500
C4—C5	1.366 (16)	N13—C111^i^	1.27 (4)
C4—H4	0.9500	C209—C210^i^	1.40 (3)
C5—N1	1.370 (16)	C209—C210	1.40 (3)
C5—C6	1.480 (15)	C210—C211	1.29 (4)
C6—N2	1.353 (13)	C210—H210	0.9500
C6—C7	1.387 (18)	C211—N23	1.46 (3)
C7—C8	1.364 (16)	C211—H211	0.9500
C7—H7	0.9500	N23—C211^i^	1.46 (3)
C8—C7^i^	1.364 (16)	P1—F14	1.594 (7)
C8—C109	1.50 (2)	P1—F16	1.594 (7)
C8—C209	1.50 (2)	P1—F11	1.594 (7)
C12—C13	1.38 (2)	P1—F13	1.594 (7)
C12—N5	1.380 (16)	P1—F12	1.594 (7)
C12—H12	0.9500	P1—F15	1.594 (7)
C13—C14	1.34 (2)	P2—F22	1.54 (2)
C13—H13	0.9500	P2—F26	1.557 (19)
C14—C15	1.42 (2)	P2—F21	1.578 (19)
C14—H14	0.9500	P2—F24	1.62 (2)
C15—C16	1.386 (18)	P2—F23	1.634 (19)
C15—H15	0.9500	P2—F25	1.644 (19)
C16—N5	1.326 (16)	O90—C90	1.209 (4)
C16—C17	1.517 (17)	C91—C90	1.481 (4)
C17—C18	1.344 (18)	C91—H91A	0.9800
C17—N4	1.361 (15)	C91—H91B	0.9800
C18—C19	1.408 (16)	C91—H91C	0.9800
C18—H18	0.9500	C90—C92	1.482 (4)
C19—C18^i^	1.408 (16)	C92—H92A	0.9800
C19—C20	1.44 (3)	C92—H92B	0.9800
C20—C21^i^	1.437 (17)	C92—H92C	0.9800
C20—C21	1.437 (17)		
			
N1—Mn1—N1^i^	144.8 (5)	C6^i^—N2—C6	119.3 (14)
N1—Mn1—N2	72.4 (3)	C6^i^—N2—Mn1	120.4 (7)
N1—Mn1—N4	107.6 (3)	C6—N2—Mn1	120.4 (7)
N1—Mn1—N5	93.5 (4)	C17^i^—N4—C17	118.3 (15)
N1—Mn1—N5^i^	97.0 (4)	C17^i^—N4—Mn1	120.9 (7)
N2—Mn1—N4	180.0	C17—N4—Mn1	120.9 (7)
N2—Mn1—N5	107.7 (3)	C16—N5—C12	118.6 (12)
N4—Mn1—N5	72.3 (3)	C16—N5—Mn1	117.6 (8)
N5—Mn1—N5^i^	144.7 (5)	C12—N5—Mn1	123.4 (9)
N2—Mn1—N1^i^	72.4 (3)	C22—N6—C22^i^	117.6 (17)
N4—Mn1—N1^i^	107.6 (3)	C110—C109—C110^i^	116 (3)
N2—Mn1—N5^i^	107.7 (3)	C110—C109—C8	121.9 (14)
N4—Mn1—N5^i^	72.3 (3)	C110^i^—C109—C8	121.9 (14)
N1^i^—Mn1—N5^i^	93.5 (4)	C111—C110—C109	115 (3)
N1—C1—C2	122.8 (14)	C111—C110—H110	122.5
N1—C1—H1	118.6	C109—C110—H110	122.5
C2—C1—H1	118.6	N13—C111—C110	132 (3)
C3—C2—C1	117.5 (14)	N13—C111—H111	114.1
C3—C2—H2	121.3	C110—C111—H111	114.1
C1—C2—H2	121.3	C111—N13—C111^i^	110 (3)
C4—C3—C2	120.9 (14)	C210^i^—C209—C210	125 (3)
C4—C3—H3	119.6	C210^i^—C209—C8	117.3 (13)
C2—C3—H3	119.6	C210—C209—C8	117.3 (13)
C5—C4—C3	119.2 (13)	C211—C210—C209	114 (3)
C5—C4—H4	120.4	C211—C210—H210	123.1
C3—C4—H4	120.4	C209—C210—H210	123.1
C4—C5—N1	121.8 (12)	C210—C211—N23	131 (3)
C4—C5—C6	124.3 (12)	C210—C211—H211	114.7
N1—C5—C6	113.9 (11)	N23—C211—H211	114.7
N2—C6—C7	120.3 (11)	C211^i^—N23—C211	106 (3)
N2—C6—C5	114.4 (11)	F14—P1—F16	90.00 (6)
C7—C6—C5	125.2 (10)	F14—P1—F11	90.00 (6)
C8—C7—C6	121.3 (12)	F16—P1—F11	90.00 (6)
C8—C7—H7	119.4	F14—P1—F13	90.00 (6)
C6—C7—H7	119.4	F16—P1—F13	90.00 (6)
C7^i^—C8—C7	117.5 (16)	F11—P1—F13	180.00 (10)
C7^i^—C8—C109	121.2 (8)	F14—P1—F12	180.0 (3)
C7—C8—C109	121.2 (8)	F16—P1—F12	90.00 (6)
C7^i^—C8—C209	121.2 (8)	F11—P1—F12	90.00 (6)
C7—C8—C209	121.2 (8)	F13—P1—F12	90.00 (6)
C13—C12—N5	119.9 (14)	F14—P1—F15	90.00 (6)
C13—C12—H12	120.1	F16—P1—F15	180.0 (10)
N5—C12—H12	120.1	F11—P1—F15	90.00 (6)
C14—C13—C12	121.8 (14)	F13—P1—F15	90.00 (6)
C14—C13—H13	119.1	F12—P1—F15	90.00 (6)
C12—C13—H13	119.1	F22—P2—F26	92.5 (11)
C13—C14—C15	119.1 (15)	F22—P2—F21	105.8 (12)
C13—C14—H14	120.5	F26—P2—F21	91.9 (10)
C15—C14—H14	120.5	F22—P2—F24	168.3 (12)
C16—C15—C14	116.9 (15)	F26—P2—F24	89.7 (11)
C16—C15—H15	121.5	F21—P2—F24	85.7 (10)
C14—C15—H15	121.5	F22—P2—F23	86.1 (11)
N5—C16—C15	123.6 (12)	F26—P2—F23	90.6 (11)
N5—C16—C17	116.1 (11)	F21—P2—F23	167.8 (12)
C15—C16—C17	120.3 (13)	F24—P2—F23	82.4 (10)
C18—C17—N4	122.1 (12)	F22—P2—F25	89.0 (11)
C18—C17—C16	125.6 (12)	F26—P2—F25	178.5 (12)
N4—C17—C16	112.2 (11)	F21—P2—F25	87.5 (10)
C17—C18—C19	120.6 (13)	F24—P2—F25	88.9 (10)
C17—C18—H18	119.7	F23—P2—F25	89.7 (10)
C19—C18—H18	119.7	C90—C91—H91A	109.5
C18^i^—C19—C18	116.3 (16)	C90—C91—H91B	109.5
C18^i^—C19—C20	121.8 (8)	H91A—C91—H91B	109.5
C18—C19—C20	121.8 (8)	C90—C91—H91C	109.5
C21^i^—C20—C21	115.5 (18)	H91A—C91—H91C	109.5
C21^i^—C20—C19	122.2 (9)	H91B—C91—H91C	109.5
C21—C20—C19	122.2 (9)	O90—C90—C91	122 (3)
C22—C21—C20	119.7 (15)	O90—C90—C92	118 (2)
C22—C21—H21	120.1	C91—C90—C92	120 (3)
C20—C21—H21	120.1	C90—C92—H92A	109.5
N6—C22—C21	123.7 (15)	C90—C92—H92B	109.5
N6—C22—H22	118.1	H92A—C92—H92B	109.5
C21—C22—H22	118.1	C90—C92—H92C	109.5
C1—N1—C5	117.7 (11)	H92A—C92—H92C	109.5
C1—N1—Mn1	123.3 (9)	H92B—C92—H92C	109.5
C5—N1—Mn1	118.9 (8)		
			
N1—C1—C2—C3	−2 (2)	C4—C5—N1—C1	−2 (2)
C1—C2—C3—C4	1 (2)	C6—C5—N1—C1	178.3 (11)
C2—C3—C4—C5	−1 (2)	C4—C5—N1—Mn1	179.1 (10)
C3—C4—C5—N1	1 (2)	C6—C5—N1—Mn1	−0.5 (15)
C3—C4—C5—C6	−179.0 (12)	C7—C6—N2—C6^i^	0.0 (8)
C4—C5—C6—N2	−179.5 (11)	C5—C6—N2—C6^i^	−179.6 (11)
N1—C5—C6—N2	0.1 (15)	C7—C6—N2—Mn1	180.0 (8)
C4—C5—C6—C7	1 (2)	C5—C6—N2—Mn1	0.4 (11)
N1—C5—C6—C7	−179.5 (12)	C18—C17—N4—C17^i^	0.2 (8)
N2—C6—C7—C8	0.1 (17)	C16—C17—N4—C17^i^	−177.4 (11)
C5—C6—C7—C8	179.6 (10)	C18—C17—N4—Mn1	−179.8 (9)
C6—C7—C8—C7^i^	0.0 (8)	C16—C17—N4—Mn1	2.6 (11)
C6—C7—C8—C109	180.0 (8)	C15—C16—N5—C12	−3.8 (19)
C6—C7—C8—C209	180.0 (8)	C17—C16—N5—C12	177.4 (10)
N5—C12—C13—C14	1 (2)	C15—C16—N5—Mn1	169.0 (10)
C12—C13—C14—C15	−1 (2)	C17—C16—N5—Mn1	−9.8 (13)
C13—C14—C15—C16	−1 (2)	C13—C12—N5—C16	1.5 (17)
C14—C15—C16—N5	4 (2)	C13—C12—N5—Mn1	−170.9 (9)
C14—C15—C16—C17	−177.6 (12)	C21—C22—N6—C22^i^	0.4 (14)
N5—C16—C17—C18	−172.7 (12)	C7^i^—C8—C109—C110	−64.7 (18)
C15—C16—C17—C18	8.5 (19)	C7—C8—C109—C110	115.3 (18)
N5—C16—C17—N4	4.9 (14)	C7^i^—C8—C109—C110^i^	115.3 (18)
C15—C16—C17—N4	−174.0 (10)	C7—C8—C109—C110^i^	−64.7 (18)
N4—C17—C18—C19	−0.5 (17)	C110^i^—C109—C110—C111	2 (2)
C16—C17—C18—C19	176.9 (10)	C8—C109—C110—C111	−178 (2)
C17—C18—C19—C18^i^	0.2 (8)	C109—C110—C111—N13	−5 (6)
C17—C18—C19—C20	−179.8 (8)	C110—C111—N13—C111^i^	3 (3)
C18^i^—C19—C20—C21^i^	40.0 (9)	C7^i^—C8—C209—C210^i^	61.4 (18)
C18—C19—C20—C21^i^	−140.0 (9)	C7—C8—C209—C210^i^	−118.6 (18)
C18^i^—C19—C20—C21	−140.0 (9)	C7^i^—C8—C209—C210	−118.6 (18)
C18—C19—C20—C21	40.0 (9)	C7—C8—C209—C210	61.4 (18)
C21^i^—C20—C21—C22	0.4 (12)	C210^i^—C209—C210—C211	−2 (3)
C19—C20—C21—C22	−179.6 (12)	C8—C209—C210—C211	178 (3)
C20—C21—C22—N6	−1 (3)	C209—C210—C211—N23	5 (6)
C2—C1—N1—C5	3 (2)	C210—C211—N23—C211^i^	−3 (3)
C2—C1—N1—Mn1	−178.6 (10)		

Symmetry code: (i) −*x*+1, *y*, −*z*+3/2.

### (2) Bis[4'-(pyridin-4-yl)-2,2':6',2''-terpyridine]manganese(II) bis(hexafluoridophosphate) acetone monosolvate Hydrogen-bond geometry (Å, º)

**Table:**

*D*—H···*A*	*D*—H	H···*A*	*D*···*A*	*D*—H···*A*
C12—H12···F25^ii^	0.95	2.31	3.23 (3)	162
C15—H15···O90^ii^	0.95	2.58	3.53 (3)	177
C18—H18···O90^iii^	0.95	2.57	3.50 (2)	168
C18—H18···O90^ii^	0.95	2.53	3.47 (2)	169
C22—H22···F21^iv^	0.95	2.47	3.42 (2)	175

Symmetry codes: (ii) *x*−1/2, −*y*+3/2, −*z*+1; (iii) *x*−1/2, *y*+1/2, *z*; (iv) −*x*+1, −*y*+2, *z*+1/2.

## References

[bb1] Beves, J. E., Bray, D. J., Clegg, J. K., Constable, E. C., Housecroft, C. E., Jolliffe, K. A., Kepert, C. J., Lindoy, L. F., Neuburger, M., Price, D. J., Schaffner, S. & Schaper, F. (2008). *Inorg. Chim. Acta*, **361**, 2582–2590.

[bb2] Beves, J. E., Constable, E. C., Decurtins, S., Dunphy, E. L., Housecroft, C. E., Keene, T. D., Neuburger, M., Schaffner, S. & Zampese, J. A. (2009). *CrystEngComm*, **11**, 2406–2416.

[bb3] Beves, J. E., Constable, E. C., Housecroft, C. E., Kepert, C. J., Neuburger, M., Price, D. J. & Schaffner, S. (2007). *CrystEngComm*, **9**, 1073–1077.

[bb4] Beves, J. E., Constable, E. C., Housecroft, C. E., Kepert, C. J. & Price, D. J. (2007). *CrystEngComm*, **9**, 456–459.

[bb5] Beves, J. E., Constable, E. C., Housecroft, C. E., Neuburger, M. & Schaffner, S. (2008). *CrystEngComm*, **10**, 344–348.

[bb6] Beves, J. E., Dunphy, E. L., Constable, E. C., Housecroft, C. E., Kepert, C. J., Neuburger, M., Price, D. J. & Schaffner, S. (2008). *Dalton Trans.* pp. 386–396. 10.1039/b714970k 18411848

[bb7] Bruker (2007). *APEX2*, *SAINT* and *SADABS*. Bruker AXS Inc., Madison, Wisconsin, USA.

[bb8] Cave, G. W. V. & Raston, C. L. (2001). *J. Chem. Soc. Perkin Trans. 1*, pp. 3258–3264.

[bb9] Constable, E. C., Housecroft, C. E., Neuburger, M., Phillips, D., Raithby, P. R., Schofield, E., Sparr, E., Tocher, D. A., Zehnder, M. & Zimmermann, Y. (2000). *Dalton Trans.* pp. 2219–2228.

[bb10] Constable, E. C., Housecroft, C. E., Neuburger, M., Schaffner, S. & Schaper, F. (2006). *Inorg. Chem. Commun.* **9**, 616–619.

[bb11] Ding, Y., Wang, F., Ku, Z.-J., Wang, L.-S. & Zhou, H.-B. (2009). *J. Struct. Chem.* **50**, 1212–1215.

[bb12] Groom, C. R. & Allen, F. H. (2014). *Angew. Chem. Int. Ed.* **53**, 662–671. 10.1002/anie.201306438 24382699

[bb13] Halper, S. R., Do, L., Stork, J. R. & Cohen, S. M. (2006). *J. Am. Chem. Soc.* **128**, 15255–15268. 10.1021/ja0645483 17117878

[bb14] Indumathy, R., Radhika, S., Kanthimathi, M., Weyhermuller, T. & Unni Nair, B. (2007). *J. Inorg. Biochem.* **101**, 434–443. 10.1016/j.jinorgbio.2006.11.002 17208305

[bb15] Kitagawa, S., Noro, S. & Nakamura, T. (2006). *Chem. Commun.* pp. 701–707. 10.1039/b511728c 16465313

[bb16] Liu, J.-J., Lin, Y.-J. & Jin, G.-X. (2014). *Organometallics*, **33**, 1283–1290.

[bb17] Mehrani, A., Morsali, A. & Ebrahimpour, P. (2013). *J. Coord. Chem.* **66**, 856–867.

[bb18] Morsali, A., Monfared, H. H., Ramazani, A., Noshiranzadeh, N., Morsali, A. & Zeller, M. (2009). *J. Coord. Chem.* **62**, 2631–2640.

[bb19] Noro, S.-I., Miyasaka, H., Kitagawa, S., Wada, T., Okubo, T., Yamashita, M. & Mitani, T. (2005). *Inorg. Chem. Commun.* **44**, 133–146. 10.1021/ic049550e 15627369

[bb20] Parsons, S., Flack, H. D. & Wagner, T. (2013). *Acta Cryst.* B**69**, 249–259. 10.1107/S2052519213010014 PMC3661305 23719469

[bb21] Paul, J., Spey, S., Adams, H. & Thomas, J. A. (2004). *Inorg. Chim. Acta*, **357**, 2827–2832.

[bb22] Pitarch López, J., Kraus, W., Reck, G., Thünemann, A. & Kurth, D. G. (2005). *Inorg. Chem. Commun.* **8**, 281–284.

[bb23] Sheldrick, G. M. (2008). *Acta Cryst.* A**64**, 112–122. 10.1107/S0108767307043930 18156677

[bb24] Sheldrick, G. M. (2015). *Acta Cryst.* C**71**, 3–8.

[bb25] Yoshida, J., Nishikiori, S.-I. & Kuroda, R. (2009). *Bull. Chem. Soc. Jpn*, **82**, 1377–1385.

